# Phylogenetic incongruence in an Asiatic species complex of the genus *Caryodaphnopsis* (Lauraceae)

**DOI:** 10.1186/s12870-024-05050-3

**Published:** 2024-06-28

**Authors:** Shiting Yang, Jiepeng Huang, Yaya Qu, Di Zhang, Yunhong Tan, Shujun Wen, Yu Song

**Affiliations:** 1 Key Laboratory of Ecology of Rare and Endangered Species and Environmental Protection (Ministry of Education) and Guangxi Key Laboratory of Landscape Resources Conservation and Sustainable Utilization in Lijiang River Basin, Guangxi Normal University, Guilin, 541004 Guangxi China; 2 Southwest Forestry University, Kunming, 650224 Yunnan China; 3 Southeast Asia Biodiversity Research Institute, Chinese Academy of Sciences & Center for Integrative Conservation, Xishuangbanna Tropical Botanical Garden, Chinese Academy of Sciences, Menglun, Mengla, Yunnan 666303 China; 4 Guangxi Key Laboratory of Plant Conservation and Restoration Ecology in Karst Terrain, Guangxi Zhuang Autonomous Region and Chinese Academy of Sciences, Guangxi Institute of Botany, Guilin, 541006 China

**Keywords:** Phylogenetic incongruence, Species complex, Tropical tree, Mitochondrial genome, Plastome, nrDNA

## Abstract

**Background:**

*Caryodaphnopsis*, a group of tropical trees (*ca*. 20 spp.) in the family Lauraceae, has an amphi-Pacific disjunct distribution: ten species are distributed in Southeast Asia, while eight species are restricted to tropical rainforests in South America. Previously, phylogenetic analyses using two nuclear markers resolved the relationships among the five species from Latin America. However, the phylogenetic relationships between the species in Asia remain poorly known.

**Results:**

Here, we first determined the complete mitochondrial genome (mitogenome), plastome, and the nuclear ribosomal cistron (nrDNA) sequences of *C. henryi* with lengths of 1,168,029 bp, 154,938 bp, and 6495 bp, respectively. We found 2233 repeats and 368 potential SSRs in the mitogenome of *C. henryi* and 50 homologous DNA fragments between its mitogenome and plastome. Gene synteny analysis revealed a mass of rearrangements in the mitogenomes of *Magnolia biondii*, *Hernandia nymphaeifolia*, and *C. henryi* and only six conserved clustered genes among them. In order to reconstruct relationships for the ten *Caryodaphnopsis* species in Asia, we created three datasets: one for the mitogenome (coding genes and ten intergenic regions), another for the plastome (whole genome), and the other for the nuclear ribosomal cistron. All of the 22 *Caryodaphnopsis* individuals were divided into four, five, and six different clades in the phylogenies based on mitogenome, plastome, and nrDNA datasets, respectively.

**Conclusions:**

The study showed phylogenetic conflicts within and between nuclear and organellar genome data of *Caryodaphnopsis* species. The sympatric *Caryodaphnopsis* species in Hekou and Malipo SW China may be related to the incomplete lineage sorting, chloroplast capture, and/or hybridization, which mixed the species as a complex in their evolutionary history.

**Supplementary Information:**

The online version contains supplementary material available at 10.1186/s12870-024-05050-3.

## Background

Trees remain a fundamental component in forest ecosystem stability with around 73,000 species and almost 20% of global plant species diversity [1]. There are an estimated 9,000 undiscovered tree species, among which roughly half to two-thirds of all is still waiting to be identified in tropical and subtropical forests [1]. These broad-leaved tree species often refer to rapid diversification and frequent introgression and compound taxonomic confusion. For instance, studies on Chinese oaks have revealed negative linear relationships between diversification rates and genetic variation, suggesting complex associations between morphological divergence and species diversification [2]. Similarly, the *Pedicularis siphonantha* complex in southwest China has shown rapid diversification, frequent introgression, and cryptic species complexes, highlighting the challenges of species delimitation based on morphological characters [3]. The phenotypes and genetic lineages of the tropical and subtropical tree species have narrowed over time in similar environments [4]. Species identification, delimitation, and description usually depend on morphological characters, but these traits often fail to distinguish the recently diverged species in tropical and subtropical forests, leading to a long and controversial debate such as species complex [4, 5].

Species complexes are a group of taxa consisting of multiple species-level lineages that cannot be reliably separated using ordinary knowledge [6]. Resolution is often hindered by their cryptic nature, making it difficult to distinguish them using traditional methods like external morphology [4, 6]. To study species complexes, a variety of methods were continuously improved, which might involve analyzing differences in individual traits, conducting reproductive isolation tests, and utilizing DNA-based techniques like molecular phylogenetics [7]. These approaches help researchers determine the boundaries between closely related organisms within a species complex. By examining the genetic, morphological, and ecological characteristics of these organisms, it is possible to identify cryptic species, hidden sibling species, and other components of a species complex [8, 9]. Recent researches have revealed that the species in difficult lineages such as bamboos, palms, oaks, rosids, and camellias [10–14], believed to be nominal species actually representing a group of closely related species, are sometimes morphologically indistinguishable.

In plants, mitochondrion and chloroplast are two DNA-containing organelles. Both have low rates of nucleotide substitution, significant variation in genome sizes, and abundant repetitive sequences [15]. Recombination is crucial for DNA replication in all organisms [16]. Mitochondrial homologous recombination essentially refers to reversible, frequent exchange of large repeats, which, if not harmful to mitochondrial function, could be retained, leading to an overall increase in mitogenome size [17]. Assembly of the mitogenomes is challenging due to their large repetitive sequences and multipartite structures. Second- together with third-generation sequencing methods help in assembling and discovering these structures [18].

Recent advances in sequencing technologies have greatly improved the acquisition of large amounts of genomic data, making it ideal for phylogenetic analysis. Plastomes, the complete DNA sequences of chloroplast, are widely utilized in phylogenetic studies in the family Lauraceae due to their ease of sequencing, assembly, and annotation [19–21]. The inclusion of the mitogenome in phylogenetic analysis has been increasingly applied in the angiosperm [22–24]. It is the diversity of genomic data that has brought the discordance of organelle and nuclear signalling into focus [25, 26]. Cytonuclear discordance refers to the incongruence between the evolutionary histories of nuclear and cytoplasmic genomes within a species or a group of species. The discordance always refers to hybridization, incomplete lineage sorting, or horizontal gene transfer [27]. Recent studies have indeed highlighted the prevalence of cytonuclear discordance in various plant species [28–30].

The amphi-Pacific genus *Caryodaphnopsis* Airy Shaw in the family Lauraceae includes about 20 tropical tree species distributed in Southeast Asia and South America [31–35]. The *Caryodaphnopsis* species in Asia differ from *Alseodaphne* and *Nothaphoebe* species by their opposite leaves, unequal filaments, and unaltered fruit pedicels, although the species in the three groups have unequal tepals and large staminodes [31, 32]. In 1940, the leaves of *C. baviensis* (Lecomte) Airy Shaw, *C. henryi* Airy Shaw, and *C. tonkinensis* (Lecomte) Airy Shaw were described as being similar to those of *Cryptocarya laevigata* Blume, while their flowers and fruits looked much like those of *Dehaasia* Blume species [31]. Recent studies have reported six new species of *Caryodaphnopsis*, including *C. laotia* Airy Shaw [36], *C. latifolia* W.T. Wang [37], *C. metalliea* Kosterm, *C. poilanei* Kosterm [32], *C. bilocellata* van der Werff & Dao [38], and *C. malipoensis* Bing Liu & Y. Yang [39]. On the other side of the Pacific Ocean, there are eight accepted species, such as *C. fosteri* van der Werff [40], *C. cogolloi* van der Werff [41], *C. tomentosa* van der Werff [42], and *C. parviflora* van der Werff [43].

*Caryodaphnopsis* has no reliable fossil record, but two molecular analyses have dated the separation between species in Asia and America to the middle Eocene (44 or 48 million years ago) [44, 45]. Both geographical groups were supported as monophyletic by previous phylogenetic analyses. The first reported chloroplast marker in *Caryodaphnopsis* was *matK*, which was used for phylogenetic analysis within the Lauraceae and suggested that *C. tonkinensis* formed a weakly supported monophyletic clade [46]. After that, Chanderbali et al. used a nuclear marker 26S ribosomal DNA sequence and four chloroplast regions (*psbA-trnH*, *rpll6*, *trnL-trnF*, and *trnT-trnL*) to recover a *Caryodaphnopsis* clade, which included *C. bilocellata* and *C. tonmentosa* [44]. Rohwer et al. (2005), using the chloroplast sequence *trnK* intron [47], found a weakly supported group comprising *C. bilocellata*, *C. tonmentosa,* and *Neocinnamomum mekongense*. Nie et al. (2007) displayed a clade comprising *C. tonmentosa* and *N. mekongense* based on ITS, *trnL-trnF*, *rpll6*, and *psbA-trnH* regions [48]. Recently, Li et al. (2016) used nuclear barcoding markers ITS and *RPB*2 and found that a monophyletic *Caryodaphnopsis* clade [45], which comprised species from two geographical groups, was strongly supported. In those phylogenetic analyses, the relationships among five South American species were well resolved, i.e., an unidentified *Caryodaphnopsis* species and its sister group containing *C. burger*, *C. fosteri*, and *C. inaequalis,* followed by *C. cogolloi* [41]. However, the relationships among species in Asia have not been resolved due to low sequence divergence in the ITS and *RPB2* markers. The separation of three individuals of *C. tonkinensis* into two branches may represent sample misidentifications or indicate intraspecific diversity [45].

In this study, we completed the assembly and annotation of the mitogenome of *C. henryi*. Data from 21 *Caryodaphnopsis* individuals in Asia were collected. The goals of this study were to (1) determine the first complete mitogenome in the Lauraceae family; (2) reveal the genomic characteristics and structural features of *C. henryi*; and (3) reconstruct the nuclear, chloroplast, and mitochondrial phylogenies of the *Caryodaphnopsis* species in Asia.

## Methods

### Plant material and geographic distributions

**Fig. 1:**
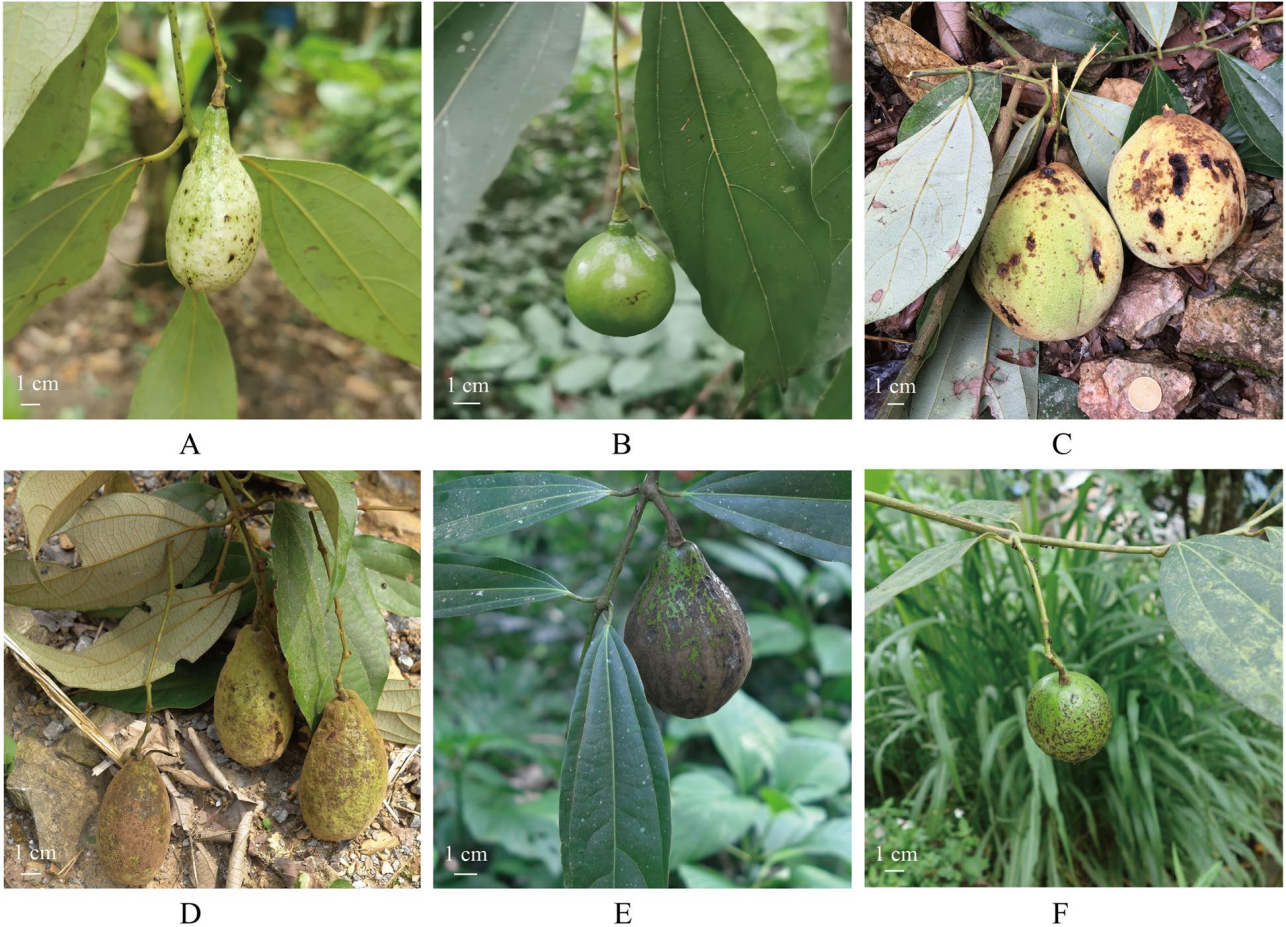
Fruits of six *Caryodaphnopsis* species (**A**: *C. tonkinensis*, **B**: *C. henryi*, **C**: *C. malipoensis*, **D**: *C. sp.* 1, **E**: *C. sp.* 2, **F**: *C. sp.* 3)

Fresh leaves and silica-gel dried materials were collected from ten *Caryodaphnopsis* species from China and Vietnam. Distribution data was compiled using herbarium records, and the voucher specimens were deposited in the Herbarium of Guangxi Normal University (Table 1). Figure 1 depicts the fruits of six *Caryodaphnopsis* species. In addition, the plastome sequence of *C. henryi* was deposited in the Lauraceae Chloroplast Genome Database (LCGDB, LAU00015, https://lcgdb.wordpress.com) [20]. And the complete mitogenome sequence of *C. henryi* was deposited in NCBI (OR987149).

**Table 1:** Sampled species of *Caryodaphnopsis* and their voucher specimens in this study

Species	Herbarium	Voucher	Geographic Origin	Latitude	Longitude
*Caryodaphnopsis laotica* Airy Shaw	GXNU	SONG Yu SY34973	Hekou, Yunnan	104.607492	23.102
*Caryodaphnopsis laotica* Airy Shaw	GXNU	SONG Yu SY36893	Hekou, Yunnan	104.048425	22.739892
*Caryodaphnopsis sp.* 1	GXNU	SONG Yu SY37063	Hekou, Yunnan	103.969734	22.708797
*Caryodaphnopsis sp.* 1	GXNU	SONG Yu SY36821	Hekou, Yunnan	104.068275	22.767897
*Caryodaphnopsis sp.* 2	GXNU	SONG Yu SY35907	Hekou, Yunnan	104.031533	22.67455
*Caryodaphnopsis sp*. 2	GXNU	SONG Yu SY34883	Hekou, Yunnan	104.032433	22.662531
*Caryodaphnopsis sp.* 3	GXNU	SONG Yu SY36729	Malipo, Yunnan	104.848122	22.969744
*Caryodaphnopsis sp.* 3	GXNU	SONG Yu SY36734	Malipo, Yunnan	104.843201	22.984373
*Caryodaphnopsis tonkinensis* (Lec.) Airy Shaw	GXNU	SONG Yu SY37141	Hekou, Yunnan	103.938156	22.672583
*Caryodaphnopsis tonkinensis* (Lec.) Airy Shaw	GXNU	SONG Yu SY36875	Hekou, Yunnan	103.938283	22.673356
*Caryodaphnopsis tonkinensis* (Lec.) Airy Shaw	GXNU	SONG Yu SY34707	Hekou, Yunnan	103.938522	22.673214
*Caryodaphnopsis tonkinensis* (Lec.) Airy Shaw	GXNU	SONG Yu SY34705	Hekou, Yunnan	103.938621	22.673386
*Caryodaphnopsis latifolia* W. T. Wang	GXNU	SONG Yu SY35963	Hekou, Yunnan	103.981613	22.714413
*Caryodaphnopsis latifolia* W. T. Wang	GXNU	SONG Yu SY35972	Hekou, Yunnan	103.969734	22.708797
*Caryodaphnopsis malipoensis* Bing Liu & Y. Yang	GXNU	SONG Yu SY36712	Malipo, Yunnan	104.848489	22.974842
*Caryodaphnopsis malipoensis* Bing Liu & Y. Yang	GXNU	SONG Yu SY36715	Malipo, Yunnan	104.828323	22.987118
*Caryodaphnopsis bilocellata* van der Werff & Dao	GXNU	SONG Yu SY37158	Hekou, Yunnan	103.960899	22.69406
*Caryodaphnopsis bilocellata* van der Werff & Dao	GXNU	SONG Yu SY35356	Hekou, Yunnan	103.960750	22.69325
*Caryodaphnopsis metallica* Kosterm	GXNU	SONG Yu SY37885	Vietnam	104.0557	22.6575
*Caryodaphnopsis henryi* Airy Shaw	GXNU	SONG Yu SY34716	Honghe, Yunnan	103.108022	23.038376
*Caryodaphnopsis henryi* Airy Shaw	GXNU	SONG Yu SY34708	Honghe, Yunnan	103.09825	23.027934

### DNA extraction and sequencing

High-quality genomic DNA of ten *Caryodaphnopsis* leaves were delivered to Tianjin Novogene Company for Illumina library preparation and second-generation sequencings. Genomic DNA was isolated from 2 g of fresh or silica-dried leaves using the CTAB technique using 4% CTAB [49], 1% PVP, and 0.2% DL dithiothreitol. The cleaved DNA fragments were tilized to build 500 bp short-insert libraries, according to the manufacturer’s handbook (Illumina). Each DNA sample received > 4.0 Gb of data from a Genome Analyzer (Illumina HiSeq 2500) at BGI-Shenzhen after being indexed by tags and pooled in one lane. A total of 27.9 Gb of sequence reads with a length of 150 bp were obtained for *C. henyri* using second-generation sequencing. Young leaves from *C. henryi* was extracted and sequenced using Oxford Nanopore PromethION platforms for third-generation sequencings. High-quality genomic DNA was extracted from the leaves using the SDS method. After library construction using SQK-LSK109 (Oxford Nanopore Technology), DNA sequencing was performed using Oxford Nanopore sequencing based on the promethION platform and 20.7 Gb of raw data with an average reads size of 27,600 bp were produced.

### Genome assembly and annotation

The unlooped mitogenome, complete plastome, and nrDNA sequences for the *Caryodaphnopsis* samples were assembled using GetOrganelle 1.7.5 [50]. To assemble a complete mitogenome of *Caryodaphnopsis henryi*, the Illumina sequencing data of *C. henryi* were initially assembled using GetOrganelle [50]. After obtaining the Nanopore third-generation sequencing reads of *C. henryi*, the adaptors were first trimmed using Porechop, and then, by aligning the trimmed reads to the scaffolds assembled by GetOrganelle using BLAST + with the parameter -evalue 1e-200 [51], the subset of long sequences that was similar to the mitochondria was obtained. Finally, these long reads and the mitochondria-related short reads that were extended by GetOrganelle were used together for hybrid assembly, which was performed by the Unicycler pipeline [52]. In the assembly result of *C. henryi*, two putative mitochondrial sequences were obtained, including a linear sequence of length 968,798 bp, and a circular sequence of length 199,231 bp. Mitogenomes were annotated using GeSeq [53], with *Liriodendron tulipifera* (KC821969) and *Magnolia biondii* (MN206019) as references. Subsequently, a detailed annotation was performed with references in Geneious Prime [54]. The circular mitogenome map was visualized using OGDRAW [55].

### Repeat and Homologous DNA Analysis

REPuter (https://bibiserv.Cebitec.uni-bielefeld.de/reputer) [56] was used to visualize forward, palindrome, reverse and complement sequences in the mitogenome of *Caryodaphnopsis henryi*, with a minimum repeat size of 30 bp, hamming distance of three and a sequence identity > 90%. And Tandem Repeats Finder (TRF) programs (https://tandem.bu.edu/) [57] was used to visualize tandem sequences with default parameters. The simple sequence repeats (SSRs) in the mitogenome of *C. henryi* was identified using MISA-web (http://pgrc.ipkgatersleben.de/misa/), with a motif size of one to six nucleotides and thresholds of 8, 5, 5, 4, 4 and 4, respectively. BLASTN was used to detect transferred DNA fragments by analysing sequence similarity between the plastome and the mitogenome, with an e-value cut-off of 1e-5. The results were visualized using the Circos module in TBtools v2.012 [58]. Mauve v.2.4.0 software was used for determining the mitogenome rearrangements among *Caryodaphnopsis henryi*, *Hernandia nymphaeifolia* (ON023262), and *Magnolia biondii* (MN206019). RAWGraphs (https://app.rawgraphs.io/) was used to describe the collinearity relationships among the gene orders in the mitogenomes of *C. henryi*, *H. nymphaeifolia*, and *M. biondii*.

### Phylogenectic analysis

All the sequence matrices were aligned with MAFFT program (version 7.31) [59] and manually modified with Geneious (version 9.1.7) [54]. Three datasets of mitochondrial, chloroplast, and nuclear ribosomal cistron sequences were comprised of the following cases: the mitochondrial dataset had 41 protein-coding genes, nine intron sequences, and ten intergenic regions; the chloroplast dataset used complete chloroplast genome sequences; and the nrDNA dataset was ETS-18S-ITS1-5.8S-ITS2-26S. A maximum likelihood (ML) analysis was carried out with IQ-TREE (version 2.1.2) [60] using 1000 ultrafast bootstrap replicates. The DNA substitution models were chosen as TVM+I+G (mtDNA), GTR+I+G (cpDNA), and GTR+G (nrDNA). The bayesian inference (BI) analysis based on the GTR+F+I (mtDNA), GTR+F+I+G4 (cpDNA), and GTR+F+I (nrDNA) models was performed with MrBayes (version 3.2.7) [61]. The BI analysis started with a random tree and sampled every 1000 generations. The first 20% of the trees was discarded as burn‐in, and the remaining trees were used to generate a majority‐rule consensus tree [62]. Visualizing and editing phylogenetic trees were performed with FigTree software (version 1.4.0).

## Results

### Organelle genome features

**Fig. 2:**
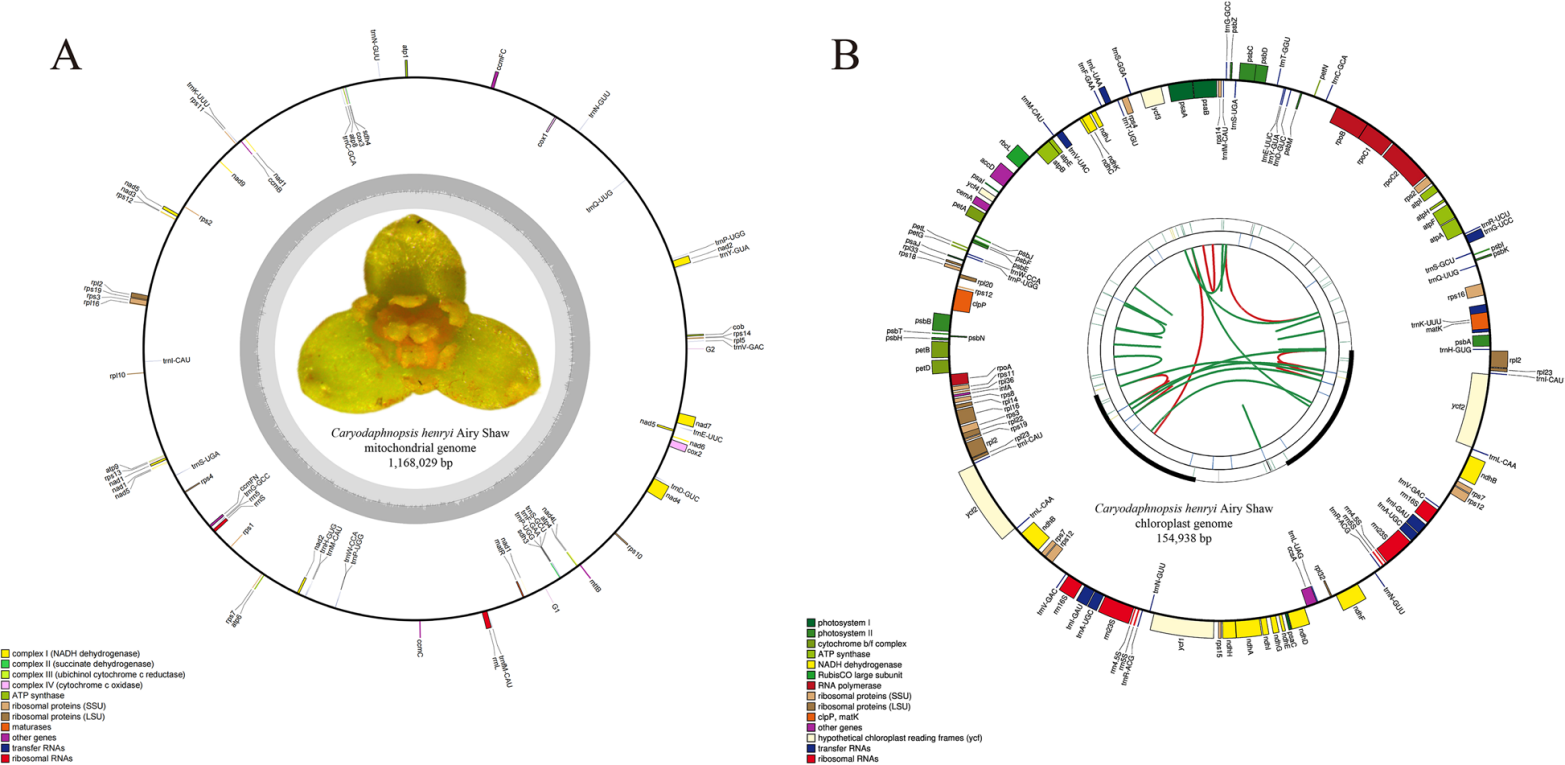
Gene maps of the *Caryodaphnopsis henryi* mitogenome (**A**) and chloroplast genome (**B**). The annotation of the genomes was performed using GeSeq. The genes that are drawn outside of the circle are transcribed clockwise, whereas those that are drawn inside the circle are transcribed counter clockwise

The DNA of *C. henryi* was extracted and sequenced using the Illumina HiSeq 2500 and Oxford Nanopore PromethION platforms for second- and third-generation sequencing, respectively. A total of 27.9 Gb raw reads of 150 bp in length and about 20.7 Gb Nanopore long read data with an average read size of 27,600 bp were used for genome assembly. We successfully assembled the whole mitogenome and chloroplast of *C. henryi* by using Illumina short reads and Nanopore long reads, which consists of one big linear contig and two tiny circular contigs with lengths of 968,798 bp, 199,231 bp, and 154,938 bp, respectively. With a total length of 1,168,029 bp, the overall base composition of the entire mitogenome is as follows: A: 26.7%, T: 26.5%, G: 23.4%, C: 23.4%, and G + C content is 46.8%. The positions of all the genes identified in the *C. henryi* mitogenome and the functional categorization of these genes are presented (Fig. 2A). The mitogenome contains 65 unique genes, including 41 protein-coding genes (PCGs), 21 transfer RNA (tRNA) genes, and 3 ribosomal RNA (rRNA) genes (Table 2). The chloroplast genome, with a length of 154,938 bp (39% G + C content), contains 113 unique genes, including 79 protein-coding genes, 30 tRNA genes, and 4 rRNA genes (Fig. 2B).

**Table 2:** Genes, separated by category, encoded by *Caryodaphnopsis henryi* mitogenome

Group of genes	Name of gene
Maturases	*matR*
Transport membrane protein	*mttB*
NADH dehydrogenase	*nad1, *nad2, nad3, *nad4, nad4L, *nad5, nad6, *nad7, nad9
ATP synthase	*atp1, atp4, atp6, atp8, atp9*
Cytochrome c biogenesis	ccmB, *ccmC, ccmFC, ccmFN
Cytochrome c oxidase	cox1, *cox2, cox3
Ubiquinol cytochrome c reductase	*cob*
Ribosomal proteins (SSU)	rps1, rps2, *rps3, rps4, rps7, *rps10, rps11, rps12, rps13, rps14,rps19
Ribosomal proteins (LSU)	*rpl2, rpl5, rpl10, rpl16
Succinate dehydrogenase	*sdh3, sdh4*
Ribosomal RNA	*rrn5, rrnL, rrnS*
Transfer RNA	*trnC-GCA, trnD-GUC, trnE-UUC, trnF-GAA, trnfM-CAU, trnG-GCC, trnH-GUG, trnI-CAU, trnK-UUU, trnM-CAU, trnN-GUU, trnN-GUU, trnP-UGG, trnP-UGG, trnP-UGG, trnQ-UUG, trnS-GCU, rnS-UGA, trnV-GAC, trnW-CCA, trnY-GUA*

A single asterisk (*) preceding gene names indicate intron-containing genes

### Repeat elements and DNA transfer analysis

**Fig. 3:**
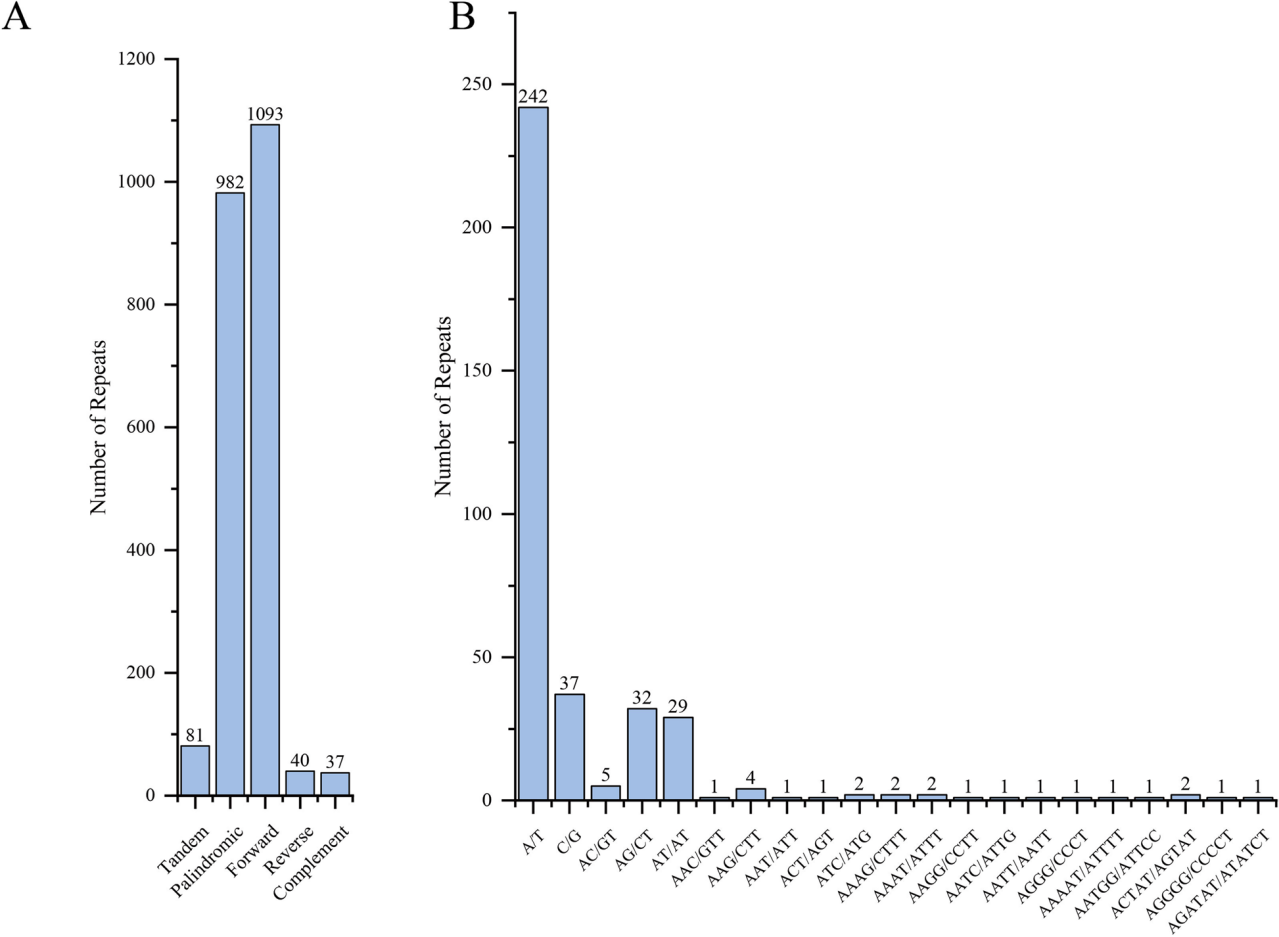
Number and distribution of long repeats (**A**) and SSRs (**B**) in mitogenome sequence of *Caryodaphnopsis henryi*

In the mitogenome of *C. henryi*, we detected 2233 repeats, and these repeats include 1093 forward repeats of 30–366 bp, 982 palindromic repeats of 30–25,242 bp, 40 reverse repeats of 30-39 bp, 37 complement repeats of 30-38bp, and 81 tandem repeats of 2–53 bp (Fig. 3A). A total of 368 potential SSRs were detected in the mitogenome of *C. henryi*, of which 279 are mononucleotides, 66 are dinucleotides, nine are trinucleotides, eight are tetranucleotides, five are pentanucleotides, and one is hexanucleotides. Of the mononucleotide repeats, A/T (86.74%) occupied the main proportion (Fig. 3B).

**Fig. 4:**
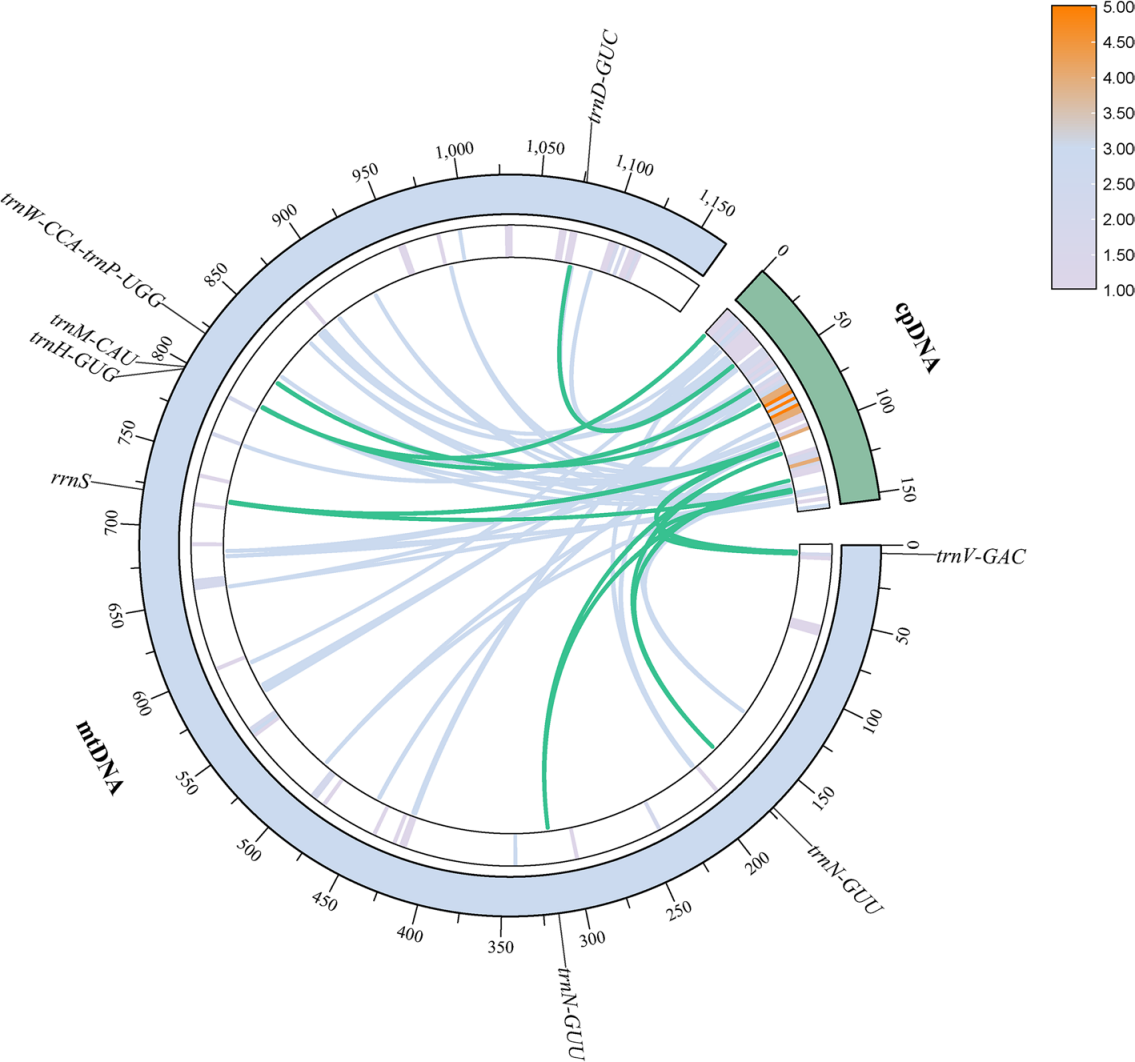
Homological sequences between mitogenome and plastome of *C. henryi*. The blue circular segment represents the mitogenome, the green circular segment represents the plastome, and the line represents the homologous fragment. Different colors in the inner circle represent gene density

The *C. henryi* mitogenome sequence was approximately 7.5 times longer than its chloroplast genome. Between the mitogenome and plastome we found a total of 50 homologous DNA fragments (Table S1, Fig. 4). The length of fragments ranged from 39 to 5262 bp. The total insert fragments were 23,583 bp in length, accounting for 2.02% of the length of mitogenome. Six tRNA genes were located in these fragments (*trnH*-GUG, *trnM*-CAU, *trnN*-GUU, *trnV*-GAC, *trnW*-CCA, *trnP*-UGG). We also detected that the fragments of chloroplast genes, such as *rrnS* and *trnD*-GUC, were located in the mitogenome.

### Mitochondrial genome comparisons

**Fig. 5:**
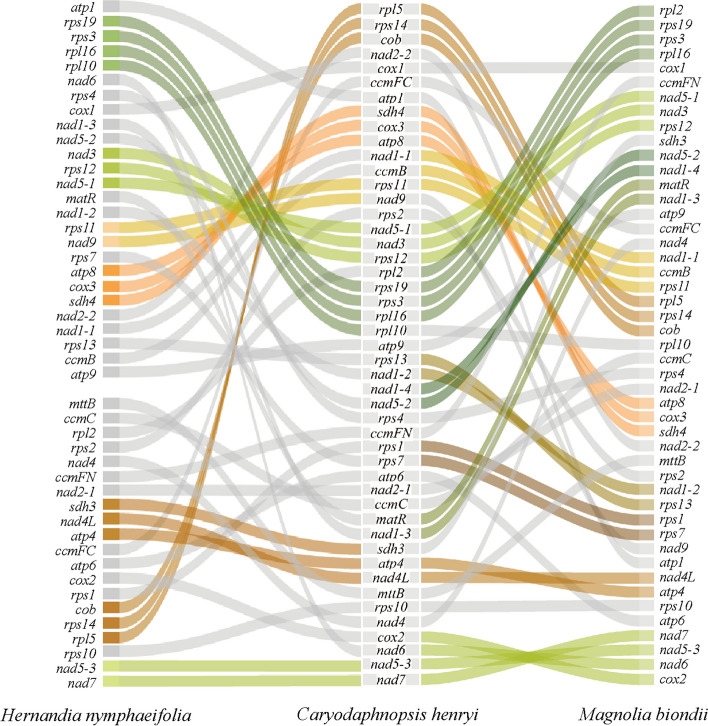
Gene order in the mitogenomes of *Hernandia nymphaeifolia*, *Magnolia biondii*, and *Caryodaphnopsis henryi*. *H. nymphaeifolia* mitochondrial genes are shown on the left, *C. henryi* mitochondrial genes in the middle, and *M. biondii* mitochondrial genes on the right, with different colors signifying the relevant collinear sections

We performed synteny and rearrangement analyses between *C. henryi* mitogenome sequences and two published mitogenome sequences of *H. nymphaeifolia* and *M. biondii*. Frequent rearrangement events were detected in both coding segments and noncoding regions (Figure S1). For the PCGs, eleven segments (Fig. 5) including *matR-nad1, nad1-ccmB-rps11, nad5-nad3-rps12, cob-rps14-rpl5, rpl2-rps19-rps3-rpl16, rps1-rps7, cox2-nad6-nad5-nad7, sdh4-cox3-atp8, atp4-nad4L, nad1-nad5,* and *rps13-nad1* were extensively conserved between *C. henryi* and* M. biondii* mitogenomes, while seven segments including *rps11-nad9*, *rps19-rps3-rpl16-rpl10, nad5-nad7, rpl5-rps14-cob, sdh4-cox3-atp8*, *nad5-nad3-rps12,* and *sdh3-atp4-nad4L* were extensively conserved between *C. henryi* and *H. nymphaeifolia* mitogenomes.

### Phylogeny of mitochondrial sequences

**Fig. 6:**
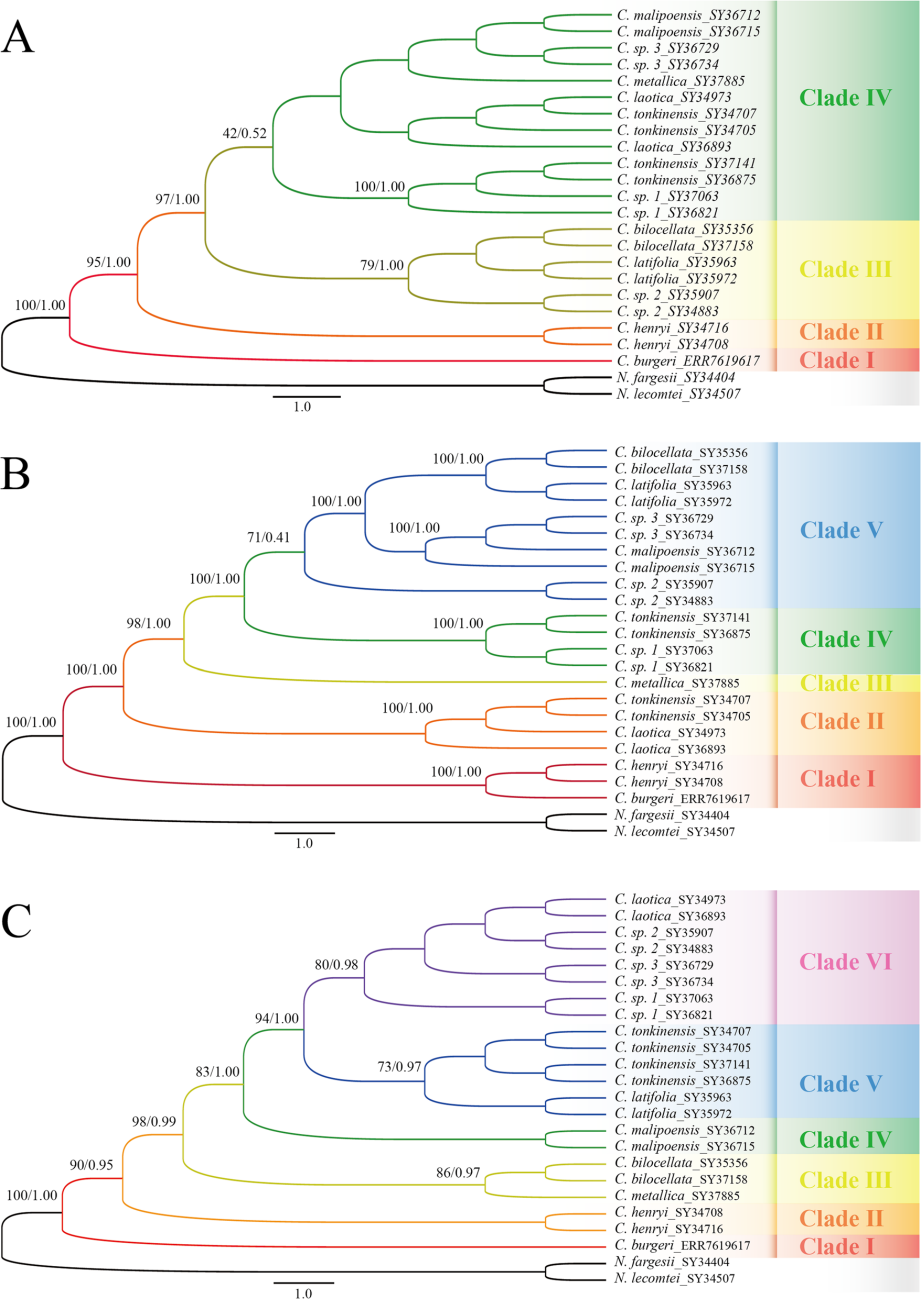
Molecular phylogenetic trees of eleven species of *Caryodaphnopsis* based on mitochondrial (**A**), complete plastomes (**B**), and nrDNA (**C**) sequences using unpartitioned Bayesian inference (BI) and maximum likelihood (ML). The trees were rooted with thesequences of *Neocinnamomum fargesii* and *N. lecomtei*. Numbers associated with the branches are ML bootstrap values (BS) and BI posterior probabilities (PP)

With the reference mitogenome of *C. henryi*, we further assembled 60 mitochondrial regions, including 41 mitochondrial protein-coding gene sequences, nine intron sequences, and ten intergenic region sequences for 21 individuals of ten *Caryodaphnopsis* species in Asia. The mitochondrial matrix (Table 3) based on the 60 regions comprises 166,057 characters, and 363 of which (depending on the consensus threshold) are parsimony-informative characters (PICs). The mitochondrial matrix was used to reconstruct phylogenetic trees, with two *Neocinnamomum* species serving as outgroups (Fig. 6A). The 22 *Caryodaphnopsis* individuals were divided into four distinct groups. The group I only included one *C. burger* individual (ML-BS = 100%, BI-PP = 1.00). The group II included two *C. henryi* individuals (ML-BS = 95%, BI-PP = 1.00). The group III included individuals of *C. bilocellata*, *C. latifolia*, and a suspected new species *C. sp.* 2 (ML-BS = 97%, BI-PP = 1.00). And the group IV included individuals of *C. laotica*, *C. malipoensis*, *C. metallica*, *C. tonkinensis*, and two suspected new species *C. sp.* 1 and *C. sp.* 3 (ML-BS = 97%, BI-PP = 1.00).

**Table 3:** The 60 mitochondrial segments were used to reconstruct the phylogenetic relationships

Types	Regions
Intergenic regions	*nad4-cox2, cox2-nad6, rps14-cob, rps7-atp6, rps13-nad1, nad5-rps4, cox3-atp8, nad1-ccmB, atp1-sdh4, nad5-nad7*
Intron regions	*ccmFC-ccmFC, cox2-cox2, nad2-nad2, nad4-nad4, nad5-nad5, nad7-nad7, rpl2-rpl2, rps3-rps3, rps10-rps10*
PCGs	*atp1, atp4, atp6, atp8, atp9, ccmB, ccmC, ccmFC, ccmFN, cob, cox1, cox2, cox3, matR, mttB, nad1, nad2, nad3, nad4, nad4L, nad5, nad6, nad7, nad9, rpl10, rpl16, rpl2, rpl5, rps1, rps10, rps11, rps12, rps13, rps14, rps19, rps2, rps3, rps4, rps7, sdh3, sdh4*

### Phylogeny of plastome sequences

The complete chloroplast genomes of 21 individuals from ten *Caryodaphnopsis* species in Asia were newly determined in the present study. They were all assembled into single circular genomes with a typical quadripartite structure, including one LSC with the lengths of 86,035 bp (*C. henryi*) to 91,966 bp (*C. sp. 2*), one SSC with the lengths of 17,310 bp (*C. sp.* 3) to 17,701 bp (*C. henryi*), and a pair of IR with the lengths of 19,694 (*C. sp.* 2) to 25,601 bp (*C. henryi*) (Table 4). The chloroplast genome alignment has 155,629 characters, 705 (0.45%) of which are PICs. The matrix of complete plastomes was used to reconstruct a phylogenetic tree of *Caryodaphnopsis* (Fig. 6B). Five well-supported groups were identified within the *Caryodaphnopsis*: the group I included one *C. burger* individual and two *C. henryi* individuals (ML-BS = 100%, BI-PP = 1.00), the group II included four individuals of *C. laotica* and *C. tonkinensis* (ML-BS = 98%, BI-PP = 1.00), the group III only included one individual of *C. metallica* (ML-BS=98%, BI-PP=1.00), the group IV included other two individuals of *C. tonkinensis* and individuals of a suspected new species *C. sp.* 1 (ML-BS = 71%, BI-PP = 0.41), and the group V included individuals of *C. bilocellata*, *C. latifolia*, *C. malipoensis*, and two suspected new species *C. sp.* 2 and *C. sp.* 3 (ML-BS = 71%, BI-PP = 0.41).

**Table 4:** Summary of ten complete plastomes of *Caryodaphnopsis*

**Types**	*C. henryi*	*C. tonkinensis*	*C. metallica*	*C. sp.3*	*C. malipoensis*	*C. bilocellata*	*C. latifolia*	*C. laotica*	*C. sp.1*	*C. sp.2*
Total cpDNA size (bp)	154,938	148,829	148,977	149,299	149,314	148,970	148,970	148,838	148,977	149,027
Length of LSC region (bp)	86,035	91,762	91,910	91,917	91,932	91,912	91,912	91,771	91,935	91,966
Length of IR region (bp)	25,601	19,695	19,700	20,036	20,036	19,695	19,695	19,695	19,695	19,694
Length of SSC region (bp)	17,701	17,677	17,667	17,310	17,311	17,668	17,668	17,677	17,652	17,673
Total GC content	39.00%	39.00%	39.10%	39.00%	39.00%	39.00%	39.00%	39.00%	39.00%	39.00%
Total number of genes (unique)	131(113)	128(113)	128(113)	128(113)	128(113)	128(113)	128(113)	128(113)	128(113)	128(113)
Protein Coding Genes	86	83	84	84	84	84	84	84	84	84
tRNA	37	36	36	36	36	36	36	36	36	36
rRNA	8	8	8	8	8	8	8	8	8	8

### Phylogeny of nuclear ribosomal cistron sequences

The nrDNA sequence of 21 individuals from ten *Caryodaphnopsis* species in Asia were newly determind in the study. The lengths of nrDNA sequences ranged from 6482 bp (*C. bilocellata*) to 6537 bp (*C. metallica*) (Table 5). Three rRNA genes and three transcribed spacers were found in these nrDNA sequences. For the 26S large-subunit rRNA (26S) region, the length varied from 3386 to 3388 bp; for the 18S small-subunit rRNA (18S) region, 1811 bp; for the 5.8S rRNA (5.8S) region, 159 bp; for the external transcribed spacer (ETS) region, from 653 to 656 bp; for the ITS1 region, from 214 to 271 bp; and for the ITS2 region, from 213 to 222 bp. The nuclear ribosomal matrix is 6,672 bp and contains 175 (2.62%) PICs. The 22 *Caryodaphnopsis* individuals were divided into six groups (Fig. 6C). The group I included the only one individual of *C. burger* (ML-BS = 100%, BI-PP = 1.00). The group II included the two individuals of *C. henryi* (ML-BS = 90%, BI-PP = 0.95). The group III included the only one individual of *C. metallica* and two individuals of *C. bilocellata* (ML-BS = 98%, BI-PP = 0.99). The group IV included the two individuals of *C. malipoensis* (ML-BS = 83%, BI-PP = 1.00). The group V included the individuals of *C. latifolia* and *C. tonkinensis* (ML-BS = 94%, BI-PP = 1.00). And the group VI included the individuals of *C. laotica* and three suspected new species *C. sp.* 1, *C. sp.* 2, and *C. sp.* 3 (ML-BS = 94%, BI-PP = 1.00).

**Table 5:** Summary of ten complete nrDNAs of *Caryodaphnopsis*

**Types**	*C. henryi*	*C. tonkinensis*	*C. metallica*	*C. sp.3*	*C. malipoensis*	*C. bilocellata*	*C. latifolia*	*C. laotica*	*C. sp.1*	*C. sp.2*
External transcribed spacer (bp)	653	653	654	654	654	656	654	654	654	654
18S small subunit rRNA (bp)	1811	1811	1811	1811	1811	1811	1811	1811	1811	1811
Internal transcribed spacer 1 (bp)	271	266	214	266	264	256	266	267	257	266
5.8S rRNA (bp)	159	159	159	159	159	159	159	159	159	159
Internal transcribed spacer 2 (bp)	213	222	222	213	213	213	215	213	213	213
26S large subunit rRNA (bp)	3388	3387	3386	3387	3387	3387	3388	3387	3387	3387

## Discussion

### General features of mitogenome

This study presents the complete mitogenome for woody plants in the family Lauraceae obtained by Illumina and Nanopore sequencing technologies (Fig. 2A). To date, there are now three orders and eight families whose mitogenomes have been sequenced within the magnoliids. The mitogenome of *Caryodaphnopsis henryi,* with a length of 1,168,029 bp, is larger than both mitogenomes of *Hernandia nymphaeifolia* and* Magnolia biondii* [63]. The length of mitochondrial genes is similar among *C. henryi, M. biondii,* and *H. nymphaeifolia*. A total of 65 mitochondrial genes in *C. henryi*, with a total length of 41,938 bp, is 800 bp smaller than those of *M. biondii* and 49 bp larger than those of *H. nymphaeifolia*. The mitochondrial intronic and intergenic regions of *C. henryi*, with a total length of 1,126,191 bp, are 201,829 bp larger than those of *M. biondii* and 632,275 bp larger than those of *H. nymphaeifolia*. There is no substantial difference in the number of mitochondrial genes, variations in noncoding DNA content are statistically linked to variations in mitogenome size [64]. In addition, siginificant length variation of mitochondrial intergenic regions has also been reported in nine species in Piperales [23].

The size variation of mitogenomes in land plants can be influenced by a variety of factors, including retrotransposon proliferation, the generation of repetitive DNA through homologous recombination, the incorporation of foreign sequences via intracellular transfer from the chloroplast or nuclear genome, or horizontal transfer of mitochondrial DNA [63, 64]. This variety has been reported in many plant species, with mitogenome sizes ranging from 66 kb in *Viscum scurruloideum* [65] to as large as 11 Mb in *Silene conica* [66]. However, in different species, the increase in mitogenome size could be caused by different factors [67].

On the one hand, a total of 2233 repeats and 368 SSRs were identified in the mitogenome of *Caryodaphnopsis henryi* (Fig. 3B). The mitogenome exhibited a significant number of dispersed repeats, primarily consisting of tandem, forward, and palindromic repeats (Fig. 3A). These repeats are critical for the recombination of the mitogenome, as they are one of the causes of variation affecting the size and structure of the mitogenome [65]. The presence of repeated sequences in the mitogenome can increase the possibility of recombination, leading to variations in the genome structure, which in turn could relate to gene expression and function [66].

On the other hand, the structure and evolutionary process of plant mitogenome make it more prone to accepting and integrating foreign DNA [64]. Horizontal gene transfer from chloroplasts to mitochondria has been reported multiple times, but the length and number of transfer fragments vary significantly between species. In this study, we found 50 homologous DNA fragments in *Caryodaphnopsis henryi *(Fig. 4), transferred from the chloroplast genome to the mitogenome. Thus, the length variation of intergenic regions might contribute to the length difference of the mitogenome in magnoliids lineages, primarily due to frequent recombination of repeated sequences and integration of foreign ones during evolution [67].

Genome rearrangement events, such as gene order changes, can reflect evolutionary distance and niche adaptation between species [68]. These events are responsible for creating extant species with conserved genes in different positions across genomes, and close species tend to have a similar set of genes or share most of them [69, 70]. Gene synteny analysis revealed a succession of rearrangements in the mitogenomes of *Magnolia biondii*, *Hernandia nymphaeifolia*, and *Caryodaphnopsis henryi* (Fig. 5). Only six gene clusters in mitogenome were found to be highly conserved across *C. henryi*, *H. nymphaeifolia*, and *M. biondii*. In addition, unlike *H. nymphaeifolia*, the mitogenomes of *C. henryi* and *M. biondii* contained eleven gene clusters. Although *C. henryi* has a closer relationship with *H. nymphaeifolia*, the number of conserved gene clusters between *C. henryi* and *H. nymphaeifolia* are less than those between *C. henryi* and *M. biondii*. The location of protein-coding genes may be poorly conserved in plant mitogenomes. The genome rearrangement events revealed by gene collinearity analysis can indeed reflect the evolutionary distance and niche adaptation between species [

71, 72].

### Cytonuclear discordance

**Fig. 7:**
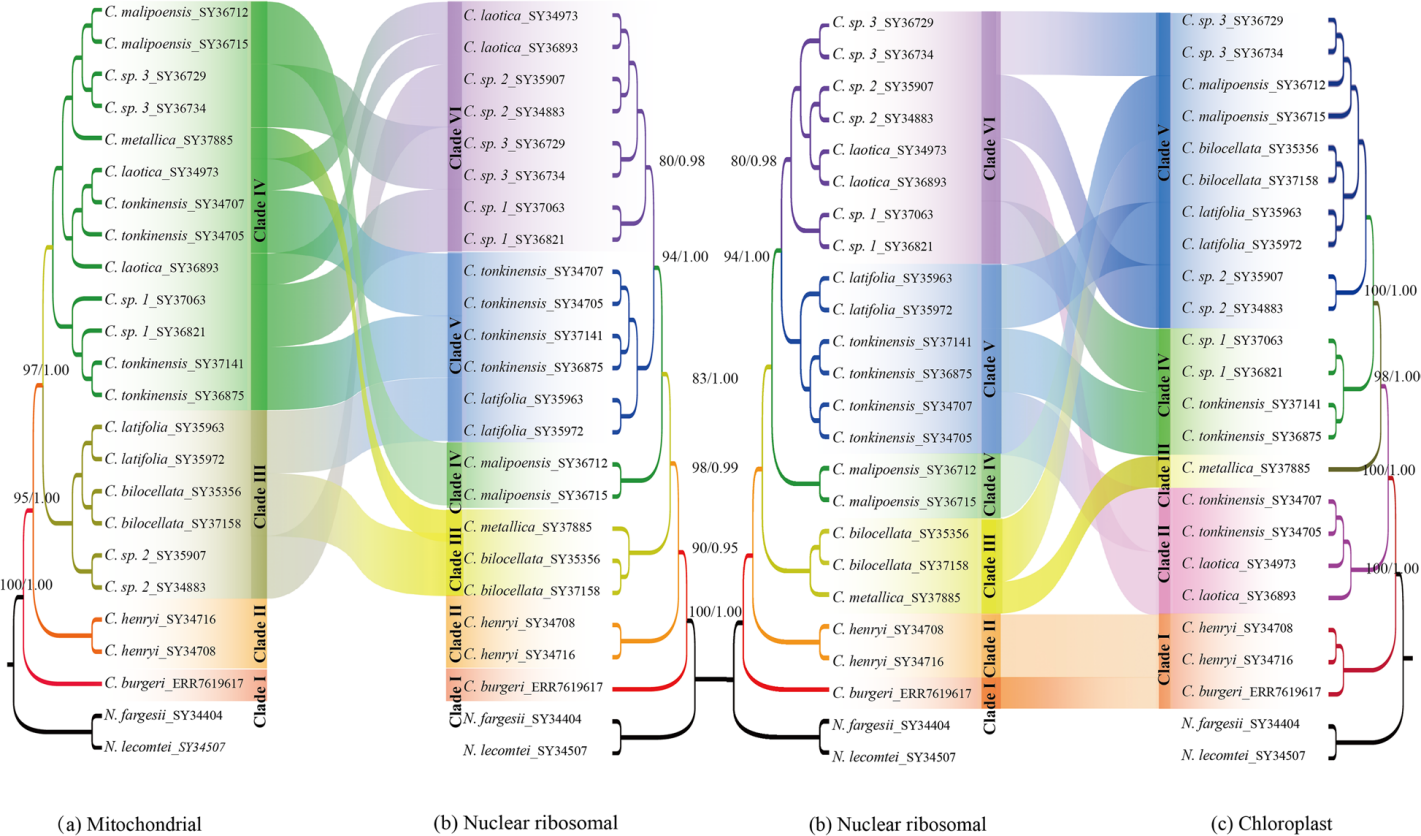
Phylogenies obtained from the three different datasets: **a** mitochondrial; **b** nuclear ribosomal; **c** chloroplast

Cytonuclear discordance, which shows markedly different phylogenetic patterns between nuclear markers and cytoplasmic genes such as mitochondrial and chloroplast genes, has been observed in various plant populations and is often attributed to processes such as hybridization and incomplete lineage sorting [30, 73, 74]. The species in the genus of *Caryodaphnopsis*, with different positions in the chloroplast and mitochondrial phylogenies relative to the nuclear phylogeny (Fig. 7). Our phylogenetic analyses revealed significant cytonuclear discordance in the genus of *Caryodaphnopsis*. The species of *C. bilocellata* and *C. metallica *are generally grouped together in the nuclear phylogeny, with different positions in the mitochondrial and chloroplast phylogenies. The species of *C. sp.* 1, *C. sp.* 2, *C. sp.* 3, and *C. laotica* are grouped together in the nuclear phylogeny, with different positions in the mitochondrial and chloroplast phylogenies. This finding is comparable with prior studies on nuclear and cytoplasmic genes inconsistencies in other plant, including in the apple genus *Malus* [

73], *balsam poplars* [30], the Australian plant genus *Adenanthos* [74]. This inconsistency may reveal complex patterns of gene flow that these species may have experienced over the course of their evolution [75]. In addition, four individuals of the *C. tonkinensis* species are clustered together in nuclear phylogeny but separated in different clades of the mitochondrial and chloroplast phylogenies. This separation of *C. tonkinensis* may represent intraspecific diversity, indicating that *Caryodaphnopsis* may have a species complex.

**Fig. 8:**
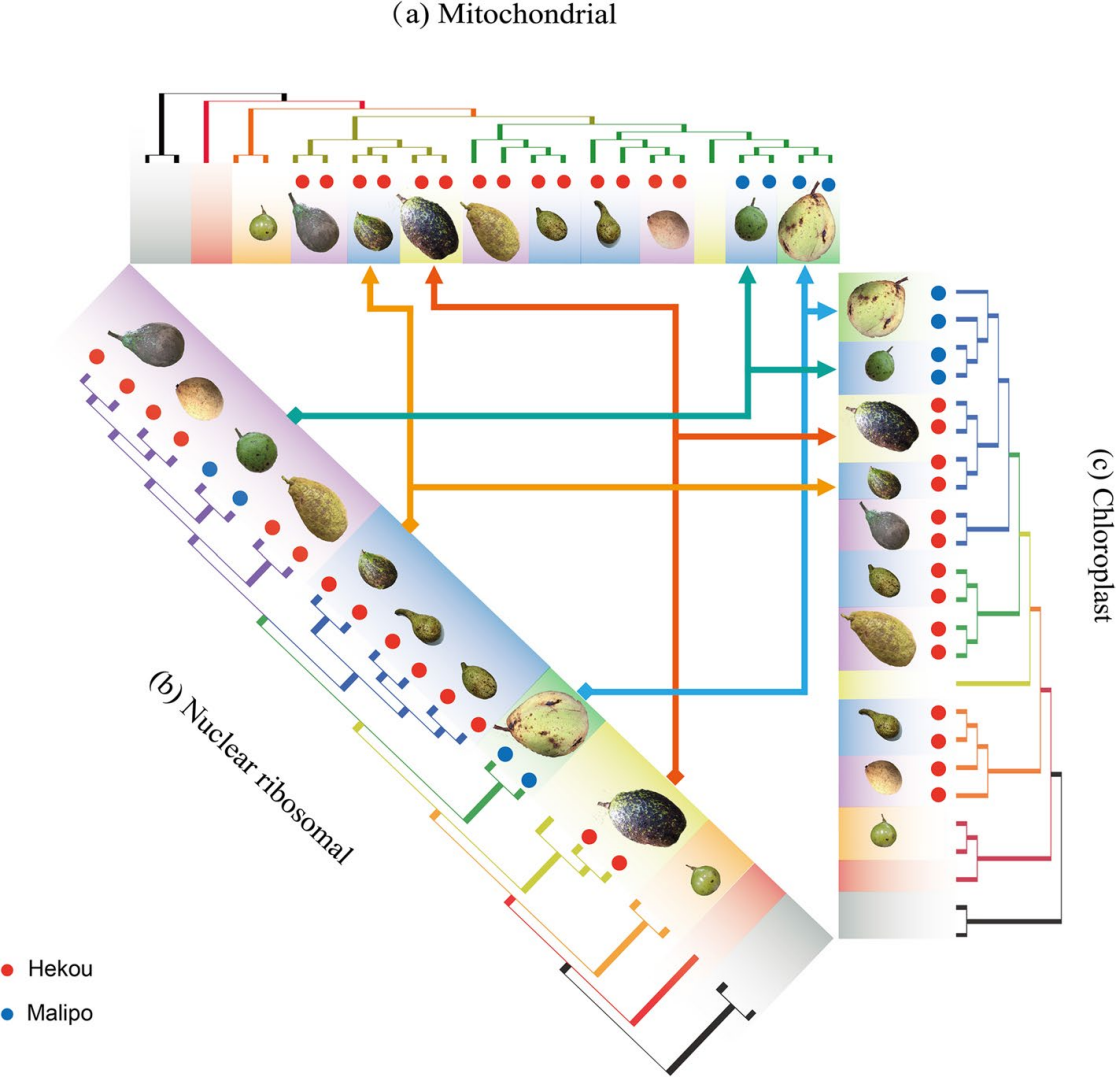
The geographic distribution and the fruit feature of *Caryodaphnopsis* are represented in the phylogenetic tree based on their mitochondrial, chloroplast, and nrDNA phylogenetic trees

Mitochondrial and chloroplast capture in plants is a phenomenon that occurs when a plant species acquires these organelles from another species through hybridization [76, 77]. In our study, the species of *Caryodaphnopsis*, with different positions in the nuclear phylogeny, are grouped together in the mitochondrial and chloroplast phylogenies. The individuals of *C. latifolia* and *C. bilocellata* collected from Hekou, China. Based on the mtDNA and cpDNA data, our phylogenomic analysis shows sisterhood of the species of *C. latifolia* and the species of *C. bilocellata*. Our phylogeny appears to reflect the organellar capture from another species. Maybe the overlap of their geographic distributions results in mitochondrial and chloroplast capture. The overlapping geographic (Fig. 8) distribution of species can lead to gene flow and hybridization, potentially resulting in gene transfer between mitochondria and chloroplasts, which can affect their clustering on the phylogenetic tree [78, 79].

**Fig. 9:**
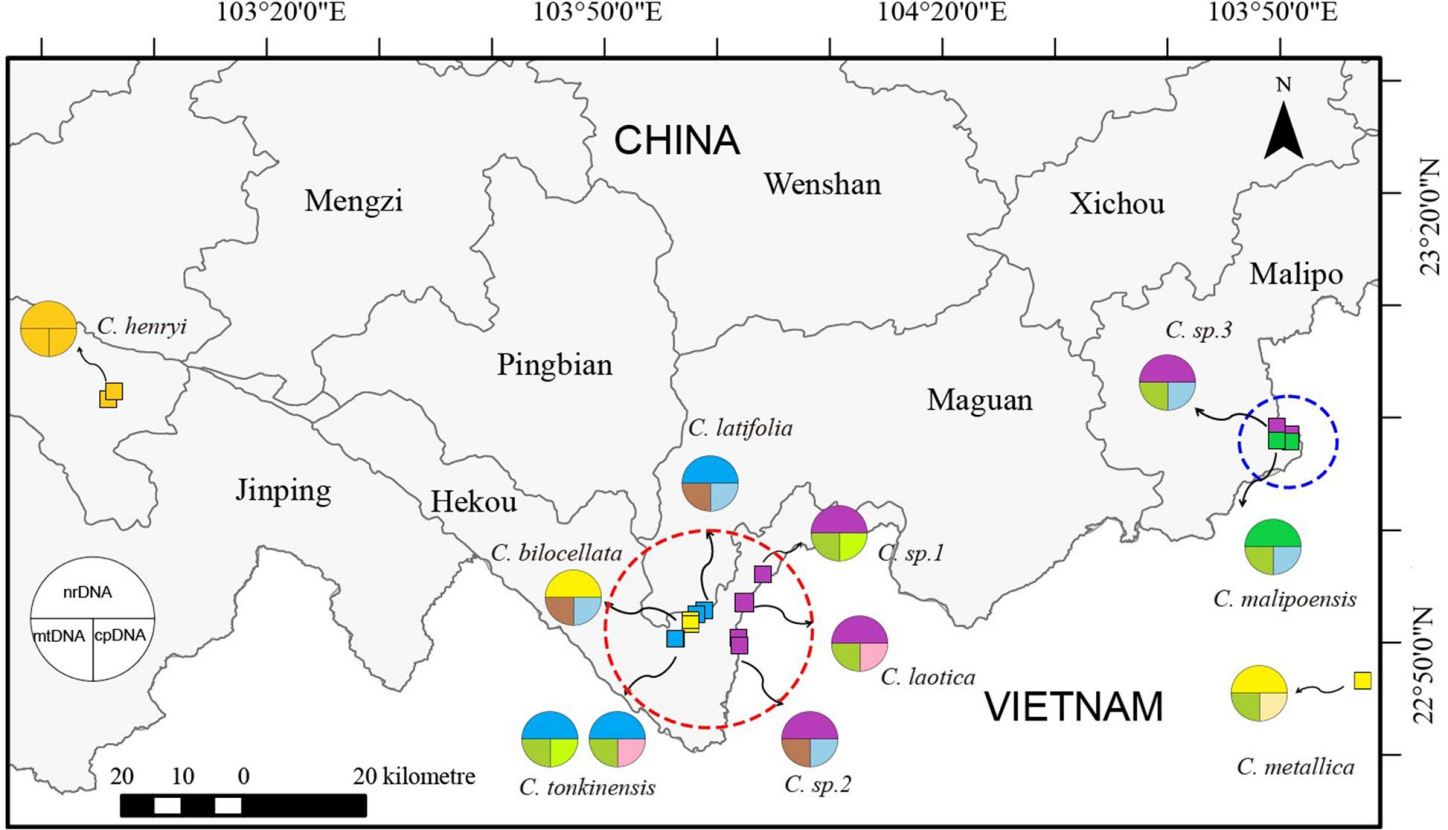
Distribution of *Caryodaphnopsis* species in this study. Each site of the species is represented by a square point. The color of the square corresponds to the species' grouping with nrDNA data. The circle is divided into three parts, representing the groupings with nrDNA, mtDNA, and cpDNA data respectively. Same color indicates the species within the consistent group of the phylogenetic topologies. The world map was downloaded from the website of the Resource and Environment Science and Data Center (http://www.resdc.cn)

Distribution sites of *Caryodaphnopsis* species shows an interesting pattern of genetic diversity across regions with comparable species richness. Malipo county of China has only two *Caryodaphnopsis* species, which share similar chloroplast and mitochondrial genomes (Fig. 9). In contrast, Hekou county of China has six *Caryodaphnopsis* species with multiple types of chloroplast, mitochondrial, and nrDNA sequences. The genetic diversity within and between species could trace implications for evolutionary processes, including adaptation to environmental stress, natural selection, and disease susceptibility [80]. The high genetic diversity observed in Hekou suggests that it may be a center of *Caryodaphnopsis* species distribution in Asia and a source of genetic resources for future conservation and breeding efforts. The phenomenon of higher genetic diversity in areas with greater species richness has been observed in other plant groups, such as tropical rainforests and alpine regions [81–83].

## Conclusions

We assembled the complete mitogenome sequence of *Caryodaphnopsis henryi*, a tropical tree in the family Lauraceae. The whole mitogenome of *C. henryi* consists of one big linear contig, with length of 968,798 bp, and one tiny circular contig, with length of 199,231 bp. The mitogenome contains 65 genes, including 41 protein-coding genes, 21 tRNA genes, and three rRNA genes. There are 50 homologous DNA fragments between the mitogenome and plastome of *C. henryi*. Comparative genomic analysis indicated that the sizes and gene orders of the three sequenced mitogenomes of *C. henryi*, *Magnolia biondii*, and *Hernandia nymphaeifolia* differed greatly. We found significant incongruence between the mitochondrial and nuclear or chloroplast phylogenies in a *Caryodaphnopsis* group. The study also revealed that *Caryodaphnopsis* species with sympatry often cluster together in the chloroplast and mitochondrial phylogenetic trees.

### Supplementary Information

**Supplementary Material 1.**

**Supplementary Material 2.**

## Data Availability

All relevant phylogenomic matrices were deposited in the the manuscript’s supplementary files. The assembled mitochondrial genome sequence was submittted to National Center for Biotechnology Information with accession number OR987149 (https://www.ncbi.nlm.nih.gov).

## Abbreviations

PCGs: Protein-Coding Genes
tRNA: Transfer RNA Genes
rRNA: Ribosomal RNA Genes
SSRs: Simple Sequence Repeats
cpDNA: Chloroplast DNA
nrDNA: Nuclear Ribosomal Cistron
mtDNA: Mitochondrial DNA
PICs: Parsimony-Informative Characters
ML: Maximum Likelihood
BI: Bayesian Inference
LSC: Large Single-Copy Region
SSC: Small Single-Copy Region
IRs: Inverted Repeats

## References

[1] Cazzolla Gatti R, Reich PB, Gamarra JGP, Crowther T, Hui C, Morera A, et al. The number of tree species on Earth. Proc Natl Acad Sci U S A. 2022;119(6):e2115329119. 10.1073/pnas.2115329119 PMC8833151 35101981

[2] Yang J, Guo YF, Chen XD, Zhang X, Ju MM, Bai GQ, et al. Framework phylogeny, evolution and complex diversification of Chinese oaks. Plants (Basel). 2020;9(8):1024. 10.3390/plants9081024 PMC7464331 32823635

[3] Liu R, Wang H, Yang JB, Corlett RT, Randle CP, Li DZ, Yu WB. Cryptic species diversification of the *Pedicularis siphonantha* complex (Orobanchaceae) in the mountains of Southwest China since the Pliocene. Front Plant Sci. 2022;13:811206. 10.3389/fpls.2022.811206 PMC8987768 35401620

[4] Pinheiro F, Dantas-Queiroz MV, Palma-Silva C. Plant species complexes as models to understand speciation and evolution: a review of South American studies. Crit Rev Plant Sci. 2018;37(1):54–80.

[5] Struck TH, Feder JL, Bendiksby M, Birkeland S, Cerca J, Gusarov VI, et al. Finding evolutionary processes hidden in cryptic species. Trends Ecol Evol. 2018;33(3):153–63. 10.1016/j.tree.2017.11.007 29241941

[6] Scherz MD, Glaw F, Hutter CR, Bletz MC, Rakotoarison A, Kohler J, Vences M. Species complexes and the importance of data deficient classification in red list assessments: the case of *Hylobatrachus* frogs. Plos One. 2019;14(8):e0219437. 10.1371/journal.pone.0219437 PMC6693689 31412043

[7] Alagona PS. Species complex: classification and conservation in American environmental history. Isis. 2016;107(4):738–61. 10.1086/689696 29897717

[8] Jorger KM, Schrodl M. How to describe a cryptic species? Practical challenges of molecular taxonomy. Front Zool. 2013;10(1):59. 10.1186/1742-9994-10-59 PMC4015967 24073641

[9] Malmstrom CM. Ecologists study the interactions of organisms and their environment. Nat Educ Knowl. 2010;3(10):88.

[10] Sijimol K, Dev SA, Sreekumar VB. DNA barcoding supports existence of morphospecies complex in endemic bamboo genus *Ochlandra* Thwaites of the Western Ghats, India. J Genet. 2020;99(1):68. 32893839

[11] Pereira DS, Hilario S, Goncalves MFM, Phillips AJL. *Diaporthe* species on palms: molecular reassessment and species boundaries delimitation in the *D. arecae* Species Complex. Microorganisms. 2023;11(11):2717. 10.3390/microorganisms11112717 PMC10673533 38004729

[12] Curtu AL, Sofletea N, Toader AV, Enescu MC. Leaf morphological and genetic differentiation between *Quercus robur* L. and its closest relative, the drought-tolerant *Quercus pedunculiflora* K. Koch. Ann Forest Sci. 2011;68:1163–72.

[13] Zhang HJ, Feng T, Landis JB, Zhang X, Meng A, Deng T, Sun H, Wang HC. Circumscription of the *Sibbaldia procumbens* complex (Potentilleae: Rosaceae) in China based on evidence from simple sequence repeat markers and morphology. Bot J Linn Soc. 2019;191(3):305–14.

[14] Li R, Yang JB, Yang SX, Li DZ. Phylogeny and taxonomy of the *Pyrenaria* complex (Theaceae) based on nuclear ribosomal ITS sequences. Nord J Bot. 2012;29:780–7.

[15] Bi CW, Lu N, Xu YQ, He CP, Lu ZH. Characterization and analysis of the mitochondrial genome of common bean (*Phaseolus vulgaris*) by comparative genomic approaches. Int J Mol Sci. 2020;21(11):3778. 10.3390/ijms21113778 PMC7312688 32471098

[16] Rocha EP, Cornet E, Michel B. Comparative and evolutionary analysis of the bacterial homologous recombination systems. Plos Genet. 2005;1(2):e15. 10.1371/journal.pgen.0010015 PMC1193525 16132081

[17] Christensen AC. Plant mitochondrial genome evolution can be explained by DNA repair mechanisms. Genome Biol Evol. 2013;5(6):1079–86. 10.1093/gbe/evt069 PMC3698917 23645599

[18] Meslier V, Quinquis B, Da Silva K, Plaza Onate F, Pons N, Roume H, Podar M, Almeida M. Benchmarking second and third-generation sequencing platforms for microbial metagenomics. Sci Data. 2022;9(1):694. 10.1038/s41597-022-01762-z PMC9652401 36369227

[19] Song Y, Yu WB, Tan YH, Jin JJ, Wang B, Yang JB, Liu B, Corlett RT. Plastid phylogenomics improve phylogenetic resolution in the Lauraceae. J Syst Evol. 2020;58(4):423–39.

[20] Song Y, Yu WB, Tan YH, Liu B, Yao X, Jin JJ, Padmanaba M, Yang JB, Corlett RT. Evolutionary comparisons of the chloroplast genome in Lauraceae and insights into loss events in the Magnoliids. Genome Biol Evol. 2017;9(9):2354–64. 10.1093/gbe/evx180 PMC5610729 28957463

[21] Song Y, Xia SW, Tan YH, Yu WB, Yao X, Xing YW, Corlett RT. Phylogeny and biogeography of the Cryptocaryeae (Lauraceae). Taxon. 2023;72(6):1244–61.

[22] Van de Paer C, Bouchez O, Besnard G. Prospects on the evolutionary mitogenomics of plants: A case study on the olive family (Oleaceae). Mol Ecol Resour. 2018;18(3):407–23. 10.1111/1755-0998.12742 29172252

[23] Yu RX, Chen XD, Long LJ, Jost M, Zhao R, Liu LM, Mower JP, dePamphilisde CW, Wanke S, Jiao YN. *De novo* assembly and comparative analyses of mitochondrial genomes in Piperales. Genome Biol Evol. 2023;15(3):evad041. 10.1093/gbe/evad041 PMC10036691 36896589

[24] Zhang X, Shan YY, Li JL, Qin QL, Yu J, Deng HP. Assembly of the complete mitochondrial genome of *Pereskia aculeata* revealed that two pairs of repetitive elements mediated the recombination of the genome. Int J Mol Sci. 2023;24(9):8366. 10.3390/ijms24098366 PMC10179450 37176072

[25] Bianconi ME, Dunning LT, Curran EV, Hidalgo O, Powell RF, Mian S, Leitch IJ, Lundgren MR, Manzi S, Vorontsova MS, et al. Contrasted histories of organelle and nuclear genomes underlying physiological diversification in a grass species. Proc Biol Sci. 2020;287(1938):20201960. 10.1098/rspb.2020.1960 PMC7735283 33171085

[26] Liu Y, Johnson MG, Cox CJ, Medina R, Devos N, Vanderpoorten A, Hedenas L, Bell NE, et al. Resolution of the ordinal phylogeny of mosses using targeted exons from organellar and nuclear genomes. Nat Commun. 2019;10(1):1485. 10.1038/s41467-019-09454-w PMC6445109 30940807

[27] Toews DPL, Brelsford A. The biogeography of mitochondrial and nuclear discordance in animals. Mol Ecol. 2012;21(16):3907–30. 10.1111/j.1365-294X.2012.05664.x 22738314

[28] Liu JX, Shi MM, Zhang ZL, Xie HB, Kong WJ, Wang QL, Zhang XX, Shi LC, et al. Phylogenomic analyses based on the plastid genome and concatenated nrDNA sequence data reveal cytonuclear discordance in genus *Atractylodes* (Asteraceae: Carduoideae). Front Plant Sci. 2022;13:1045423. 10.3389/fpls.2022.1045423 PMC9752137 36531370

[29] Lee-Yaw JA, Grassa CJ, Joly S, Andrew RL, Rieseberg LH. An evaluation of alternative explanations for widespread cytonuclear discordance in annual sunflowers (*Helianthus*). New Phytol. 2019;221(1):515–26. 10.1111/nph.15386 30136727

[30] Huang DI, Hefer CA, Kolosova N, Douglas CJ, Cronk QCB. Whole plastome sequencing reveals deep plastid divergence and cytonuclear discordance between closely related balsam poplars, *Populus balsamifera* and *P. Trichocarpa* (Salicaceae). New Phytol. 2014;204(3):693–703. 10.1111/nph.12956 25078531

[31] Airy-Shaw HK. Notes on two asiatic genera of Lauraceae. Bullet Miscell Info (Royal Gardens, Kew). 1940;2:74–7.

[32] Kostermans AJGH. A monograph of *Caryodaphnopsis* Airy Shaw. Reinwardtia. 1974;9(1):123–37.

[33] Cao ZY, Qu YY, Song Y, Xin PY. Comparative genomics and phylogenetic analysis of chloroplast genomes of Asian *Caryodaphnopsis* taxa (Lauraceae). Gene. 2024;907:148259. 10.1016/j.gene.2024.148259 38346458

[34] van der Werff H, Richter HG. *Caryodaphnopsis* Airy-Shaw (Lauraceae), a Genus New to the Neotropics. Syst Bot. 1985;10(2):166–73.

[35] Li XW, Li J. Notes on the taxonomy and distribution of the genus *Caryodaphnopsis* of Lauraceae and to discuss the characteristics of its area-type. Plant Diversity. 1991;13(01):1–3.

[36] Wu CY, Wang WT. A preliminary report on floristic studies of tropical and subtropical region of Yunnan. Acta Phytotaxonomica Sinica. 1957;6:183–254.

[37] Airy Shaw HK. A new species of *Caryodaphnopsis* (Lauraceae). Harv Pap Bot. 1960;14:250–1.

[38] van der Werff H. A new species of *Caryodaphnopsis* (Lauraceae) from Vietnam. Novon. 1999;9(4):584–6.

[39] Liu B, Yang Y, Ma K. A new species of *Caryodaphnopsis* Airy Shaw (Lauraceae) from southeastern Yunnan China. Phytotaxa. 2013;118(1):1–8.

[40] Van der Werff H. A new species of *Caryodaphnopsis* (Lauraceae) from Peru. Syst Bot. 1986;11(3):415–8.

[41] van der Werff H. Eight new species and one new combination of neotropical Lauraceae. Ann Mo Bot Gard. 1988;75(2):402–19.

[42] van der Werff H. New Species of Lauraceae from Ecuador and Peru. Ann Mo Bot Gard. 1991;78(2):409–23.

[43] Van der Werff H. A new species of *Caryodaphnopsis* (Lauraceae) from Harvard. Botany. 2012;17(1):39–41.

[44] Chanderbali AS, van der Werff H, Renner SS. Phylogeny and historical biogeography of Lauraceae: evidence from the chloroplast and nuclear genomes. Ann Mo Bot Gard. 2001;88(1):104–34.

[45] Li L, Madriñán S, Li J. Phylogeny and biogeography of *Caryodaphnopsis* (Lauraceae) inferred from lowcopy nuclear gene and ITS sequences. Taxon. 2016;65(3):433–43.

[46] Rohwer JG. Toward a phylogenetic classification of the Lauraceae: evidence from matK sequences. Syst Bot. 2000;25(1):60–71.

[47] Rohwer JG, Rudolph B. Jumping genera: The phylogenetic positions of *Cassytha*, *Hypodaphnis*, and *Neocinnamomum* (Lauraceae) based on different analyses of trnK intron sequences. Ann Missouri Bot Garden. 2005;92(2):153–78.

[48] Nie ZL, Wen J, Sun H. Phylogeny and biogeography of *Sassafras* (Lauraceae) disjunct between eastern Asia and eastern North America. Plant Syst Evol. 2007;267(1–4):191–203.

[49] Doyle JJ, Dickson EE. Preservation of plant samples for DNA restriction Endonuclease analysis. Taxon. 1987;36(4):715–22.

[50] Jin JJ, Yu WB, Yang JB, Song Y, dePamphilis CW, Yi TS, Li DZ. GetOrganelle: a fast and versatile toolkit for accurate de novo assembly of organelle genomes. Genome Biol. 2020;21(1):241. 10.1186/s13059-020-02154-5 PMC7488116 32912315

[51] Camacho C, Coulouris G, Avagyan V, Ma N, Papadopoulos J, Bealer K, Madden TL. BLAST+: architecture and applications. BMC Bioinformatics. 2009;10:421. 10.1186/1471-2105-10-421 PMC2803857 20003500

[52] Wick RR,Judd LM, Gorrie CL, Holt KE. Unicycler: resolving bacterial genome assemblies from short and long sequencing reads. Plos Comput Biol. 2017;13(6):e1005595. 10.1371/journal.pcbi.1005595 PMC5481147 28594827

[53] Tillich M, Lehwark P, Pellizzer T, Ulbricht-Jones ES, Fischer A, Bock R, Greiner S. GeSeq – versatile and accurate annotation of organelle genomes. Nucleic Acids Res. 2017;45(W1):W6–W11. 10.1093/nar/gkx391 PMC5570176 28486635

[54] Kearse M, Moir R, Wilson A, Stones-Havas S, Cheung M, Sturrock S, Buxton S, Cooper A, Markowitz S, Duran C, et al. Geneious Basic: an integrated and extendable desktop software platform for the organization and analysis of sequence data. Bioinformatics. 2012;28(12):1647–9. 10.1093/bioinformatics/bts199 PMC3371832 22543367

[55] Lohse M, Drechsel O, Bock R. OrganellarGenomeDRAW (OGDRAW): a tool for the easy generation of high-quality custom graphical maps of plastid and mitochondrial genomes. Curr Genet. 2007;52(5–6):267–74. 10.1007/s00294-007-0161-y 17957369

[56] Kurtz S, Choudhuri JV, Ohlebusch E, Schleiermacher C, Stoye J, Giegerich R. REPuter: the manifold applications of repeat analysis on a genomic scale. Nucleic Acids Res. 2001;29(22):4633–42. 10.1093/nar/29.22.4633 PMC92531 11713313

[57] Benson G. Tandem repeats finder: a program to analyze DNA sequences. Nucleic Acids Res. 1999;27(2):573–80. 10.1093/nar/27.2.573 PMC148217 9862982

[58] Chen CJ, Wu Y, Li JW, Wang X, Zeng ZH, Xu J, Liu YL, Feng JT, Chen H, He YH, Xia R. TBtools-II: A “One for all, All for one” bioinformatics platform for biological big-data mining. Mol Plant. 2023;16(11):1733-42. 10.1016/j.molp.2023.09.010 37740491

[59] Katoh K, Standley DM. MAFFT multiple sequence alignment software version 7: improvements in performance and usability. Mol Biol Evol. 2013;30(4):772–80. 10.1093/molbev/mst010 PMC3603318 23329690

[60] Nguyen LT, Schmidt HA, von Haeseler A, Minh BQ. IQ-TREE: a fast and effective stochastic algorithm for estimating maximum-likelihood phylogenies. Mol Biol Evol. 2015;32(1):268–74. 10.1093/molbev/msu300 PMC4271533 25371430

[61] Ronquist F, Huelsenbeck JP. MrBayes 3: Bayesian phylogenetic inference under mixed models. Bioinformatics. 2003;19(12):1572–4. 10.1093/bioinformatics/btg180 12912839

[62] Rambaut A, Drummond AJ, Xie D, Baele G, Suchard MA. Posterior Summarization in Bayesian Phylogenetics Using Tracer 1.7. Syst Biol. 2018;67(5):901–4. 10.1093/sysbio/syy032 PMC6101584 29718447

[63] Dong SS, Liu M, Liu Y, Chen F, Yang T, Chen L, Zhang XT, Guo X, Fang DM, Li LZ, et al. The genome of *Magnolia biondii* Pamp. provides insights into the evolution of Magnoliales and biosynthesis of terpenoids. Hortic Res. 2021;8(1):38. 10.1038/s41438-021-00471-9 PMC7917104 33642574

[64] Rice DW, Alverson AJ, Richardson AO, Young GJ, Sanchez-Puerta MV, Munzinger J, Barry K, Boore JL, et al. Horizontal transfer of entire genomes via mitochondrial fusion in the angiosperm Amborella. Science. 2013;342:1468–73. 10.1126/science.1246275 24357311

[65] Guo WH, Grewe F, Fan WS, Young GJ, Knoop V, Palmer JD, Mower JP. *Ginkgo* and *Welwitschia* mitogenomes reveal extreme contrasts in gymnosperm mitochondrial evolution. Mol Biol Evol. 2016;33(6):1448–60. 10.1093/molbev/msw024 26831941

[66] Gualberto JM, Newton KJ. Plant mitochondrial genomes: dynamics and mechanisms of mutation. Annu Rev Plant Biol. 2017;68:225–52. 10.1146/annurev-arplant-043015-112232 28226235

[67] Woloszynska M, Bocer T, Mackiewicz P, Janska H. A fragment of chloroplast DNA was transferred horizontally, probably from non-eudicots, to mitochondrial genome of *Phaseolus*. Plant Mol Biol. 2004;56(5):811–20. 10.1007/s11103-004-5183-y 15803417

[68] Siqueira G, Brito KL, Dias U, Dias Z. Heuristics for genome rearrangement distance with replicated genes. IEEE/ACM Trans Comput Biol Bioinform. 2021;18(6):2094–108. 10.1109/TCBB.2021.3095021 34232886

[69] Mao M, Gibson T, Dowton M. Evolutionary dynamics of the mitochondrial genome in the evaniomorpha (hymenoptera)-a group with an intermediate rate of gene rearrangement. Genome Biol Evol. 2014;6(7):1862–74. 10.1093/gbe/evu145 PMC4122943 25115010

[70] Zhang Y, Gong L, Lu XT, Miao ZL, Jiang LH, Liu BJ, Liu LQ, Li PF, Zhang X, Lü ZM. Comparative mitochondrial genome analysis of Varunidae and its phylogenetic implications. Acta Oceanol Sin. 2022;41(6):119–31.

[71] Du YH, Zou JR, Yin ZQ, Chen TJ. Pan-chromosome and comparative analysis of *Agrobacterium fabrum* reveal important traits concerning the genetic diversity, evolutionary dynamics, and niche adaptation of the species. Microbiol Spectr. 2023;11(2):e0292422. 10.1128/spectrum.02924-22 PMC10100860 36853054

[72] Ben Moussa H, Pedron J, Hugouvieux-Cotte-Pattat N, Barny MA. Two species with a peculiar evolution within the genus *Pectobacterium* suggest adaptation to a new environmental niche. Environ Microbiol. 2023;25(11):2465–80. 10.1111/1462-2920.16479 37550252

[73] Liu BB, Ren C, Kwak M, Hodel RGJ, Xu C, He J, Zhou WB, Huang CH, Ma H, Qian GZ, et al. Phylogenomic conflict analyses in the apple genus *Malus s.l.* reveal widespread hybridization and allopolyploidy driving diversification, with insights into the complex biogeographic history in the Northern Hemisphere. J Integr Plant Biol. 2022;64(5):1020–43. 10.1111/jipb.13246 35274452

[74] Nge FJ, Biffin E, Thiele KR, Waycott M. Reticulate evolution, ancient Chloroplast Haplotypes, and rapid radiation of the Australian plant genus *Adenanthos* (Proteaceae). Front Ecol Evol. 2021;8:616741.

[75] Mao Y, Peng TT, Shao F, Zhao QY, Peng ZG. Molecular evolution of the hemoglobin gene family across vertebrates. Genetica. 2023;151(3):201–13. 10.1007/s10709-023-00187-9 37069365

[76] Kawabe A, Nukii H, Furihata HY. Exploring the history of chloroplast capture in *Arabis* using whole chloroplast genome sequencing. Int J Mol Sci. 2018;19(2):602. 10.3390/ijms19020602 PMC5855824 29463014

[77] Baldwin E, McNair M, Leebens-Mack J. Rampant chloroplast capture in Sarracenia revealed by plastome phylogeny. Front Plant Sci. 2023;14:1237749. 10.3389/fpls.2023.1237749 PMC10497973 37711293

[78] Lin QS, Banerjee A, Stefanovic S. Mitochondrial phylogenomics of *Cuscuta* (Convolvulaceae) reveals a potentially functional horizontal gene transfer from the host. Genome Biol Evol. 2022;14(6):evac091. 10.1093/gbe/evac091 PMC9234195 35700229

[79] Filip E, Skuza L. Horizontal gene transfer involving chloroplasts. Int J Mol Sci. 2021;22(9):4484. 10.3390/ijms22094484 PMC8123421 33923118

[80] Stange M, Barrett RDH, Hendry AP. The importance of genomic variation for biodiversity, ecosystems and people. Nat Rev Genet. 2021;22(2):89–105. 10.1038/s41576-020-00288-7 33067582

[81] Kuria MW. Evaluation of genetic diversity in *Strychnos henningsii* selected from nine populations in Kenya based on RAPD markers. East Afr J Agri Biotechnol. 2023;6(1):406–21.

[82] Chen X, Feng Y, Chen S, Yang K, Wen XY, Sun Y. Species delimitation and genetic relationship of *Castanopsis hainanensis* and *Castanopsis wenchangensis* (Fagaceae). Plants (Basel). 2023;12(20):3544. 10.3390/plants12203544 PMC10609670 37896008

[83] Born C, Alvarez N, McKey D, Ossari S, Wickings EJ, Hossaert-McKey M, Chevallier MH. Insights into the biogeographical history of the lower Guinea forest domain: evidence for the role of refugia in the intraspecific differentiation of *Aucoumea klaineana*. Mol Ecol. 2011;20(1):131–42. 10.1111/j.1365-294X.2010.04919.x 21091559

