# Supplementary material for: Phylogenetic incongruence in an Asiatic species complex of the genus Caryodaphnopsis (Lauraceae)
Source: BMC Plant Biol. 2024 Jun 28;24:616. doi: 10.1186/s12870-024-05050-3 (PMC11212351; doi:10.1186/s12870-024-05050-3)
Supplement: Supplementary file 1 — Supplementary Material 1. [file 12870_2024_5050_MOESM1_ESM.docx]

**Table S1** Homological sequences between mitochondrial and chloroplast genomes of *Caryodaphnopsis* *henryi.*

| **No** | **Map Length** | **Identity%** | **Gap Option** | **CP Start Position** | **CP End Position** | **CP Region** | **Mt Start Position** | **Mt End Position** | **Mt Region** |
| --- | --- | --- | --- | --- | --- | --- | --- | --- | --- |
| 1 | 5262 | 96.066 | 26 | 571272 | 576498 | Intergenic region | 54943 | 60180 | trnM-CAU-atpE-atpB-rbcL-accD |
| 2 | 4610 | 90.824 | 41 | 870636 | 875230 | Intergenic region | 23809 | 28349 | rpoC1-ropB |
| 3 | 1549 | 99.677 | 1 | 4569 | 6117 | trnV-GAC | 100926 | 102472 | trnV-GAC-rrn16S |
| 4 | 1549 | 99.677 | 1 | 4569 | 6117 | trnV-GAC | 140048 | 138502 | rrn16S-trnV-GAC |
| 5 | 1070 | 91.121 | 9 | 419535 | 420597 | Intergenic region | 36639 | 35597 | psbC |
| 6 | 704 | 94.318 | 3 | 889735 | 890428 | Intergenic region | 11725 | 12428 | atpA |
| 7 | 718 | 92.479 | 6 | 193057 | 193762 | Intergenic region | 63258 | 62552 | ycf4 |
| 8 | 997 | 83.149 | 42 | 1077908 | 1078870 | Intergenic region | 29481 | 30445 | petN |
| 9 | 315 | 94.603 | 3 | 595573 | 595886 | Intergenic region | 13046 | 13358 | atpF |
| 10 | 346 | 91.04 | 1 | 449594 | 449934 | Intergenic region | 85325 | 85670 | rpl22 |
| 11 | 457 | 84.464 | 17 | 820527 | 820961 | trnW-CCA-trnP-UGG | 68389 | 68837 | trnW-CCA-trnP-UGG |
| 12 | 535 | 81.869 | 18 | 420744 | 421245 | Intergenic region | 35583 | 35058 | psbD |
| 13 | 330 | 89.091 | 5 | 1076613 | 1076933 | Intergenic region | 32874 | 32552 | trnY-GUA-trnE-UUC |
| 14 | 252 | 91.27 | 6 | 193770 | 194019 | Intergenic region | 14331 | 14084 | Intergenic region |
| 15 | 359 | 83.287 | 13 | 1077070 | 1077416 | trnD-GUC | 32373 | 32030 | trnD-GUC |
| 16 | 148 | 97.973 | 0 | 799352 | 799499 | Intergenic region | 102503 | 102356 | rrn16S |
| 17 | 145 | 98.621 | 0 | 799352 | 799496 | Intergenic region | 138471 | 138615 | rrn16S |
| 18 | 467 | 75.803 | 17 | 720867 | 721318 | rrnS | 102697 | 102241 | rrn16S |
| 19 | 467 | 75.803 | 17 | 720867 | 721318 | rrnS | 138277 | 138733 | rrn16S |
| 20 | 119 | 96.639 | 1 | 4210 | 4327 | Intergenic region | 100807 | 100925 | Intergenic region |
| 21 | 128 | 94.531 | 1 | 860783 | 860910 | Intergenic region | 104019 | 103893 | trnI-GAU |
| 22 | 128 | 94.531 | 1 | 860783 | 860910 | Intergenic region | 136955 | 137081 | trnI-GAU |
| 23 | 122 | 95.902 | 1 | 4210 | 4330 | Intergenic region | 140167 | 140046 | Intergenic region |
| 24 | 121 | 95.041 | 0 | 985080 | 985200 | Intergenic region | 104013 | 103893 | trnI-GAU |
| 25 | 121 | 95.041 | 0 | 985080 | 985200 | Intergenic region | 136961 | 137081 | trnI-GAU |
| 26 | 109 | 97.248 | 2 | 1093732 | 1093840 | Intergenic region | 17733 | 17627 | rpoC2 |
| 27 | 313 | 77.636 | 8 | 721416 | 721725 | rrnS | 102141 | 101834 | rrn16S |
| 28 | 313 | 77.636 | 8 | 721416 | 721725 | rrnS | 138833 | 139140 | rrn16S |
| 29 | 200 | 83 | 9 | 1077479 | 1077676 | Intergenic region | 31938 | 31750 | Intergenic region |
| 30 | 163 | 82.822 | 9 | 679891 | 680041 | Intergenic region | 94887 | 95048 | ycf2 |
| 31 | 84 | 95.238 | 0 | 172063 | 172146 | trnN-GUU | 109791 | 109708 | trnN-GUU |
| 32 | 84 | 95.238 | 0 | 172063 | 172146 | trnN-GUU | 131183 | 131266 | trnN-GUU |
| 33 | 163 | 82.822 | 9 | 679891 | 680041 | Intergenic region | 146087 | 145926 | ycf2 |
| 34 | 81 | 95.062 | 0 | 798396 | 798476 | trnH-GUG | 37 | 117 | trnH-GUG |
| 35 | 79 | 93.671 | 0 | 316012 | 316090 | trnN-GUU | 109791 | 109713 | trnN-GUU |
| 36 | 79 | 93.671 | 0 | 316012 | 316090 | trnN-GUU | 131183 | 131261 | trnN-GUU |
| 37 | 79 | 92.405 | 0 | 798979 | 799057 | trnM-CAU | 55001 | 54923 | trnM-CAU |
| 38 | 88 | 89.773 | 1 | 826660 | 826739 | Intergenic region | 105107 | 105194 | trnA-UGC |
| 39 | 88 | 89.773 | 1 | 826660 | 826739 | Intergenic region | 135867 | 135780 | trnA-UGC |
| 40 | 57 | 98.246 | 0 | 766080 | 766136 | Intergenic region | 52016 | 52072 | ndhK |
| 41 | 97 | 82.474 | 0 | 923094 | 923190 | Intergenic region | 105885 | 105981 | rrn23S |
| 42 | 97 | 82.474 | 0 | 923094 | 923190 | Intergenic region | 135089 | 134993 | rrn23S |
| 43 | 54 | 94.444 | 0 | 498671 | 498724 | Intergenic region | 101853 | 101800 | rrn16S |
| 44 | 60 | 91.667 | 0 | 498665 | 498724 | Intergenic region | 139115 | 139174 | rrn16S |
| 45 | 39 | 100 | 0 | 135926 | 135964 | Intergenic region | 103083 | 103121 | rrn16S |
| 46 | 39 | 100 | 0 | 135926 | 135964 | Intergenic region | 137891 | 137853 | rrn16S |
| 47 | 59 | 89.831 | 1 | 656532 | 656590 | Intergenic region | 99370 | 99317 | rps12 |
| 48 | 59 | 89.831 | 1 | 656532 | 656590 | Intergenic region | 141604 | 141657 | rps12 |
| 49 | 40 | 97.5 | 0 | 683953 | 683992 | Intergenic region | 93181 | 93142 | ycf2 |
| 50 | 40 | 97.5 | 0 | 683953 | 683992 | Intergenic region | 147793 | 147832 | ycf2 |
