# Supplementary figures and images for: Phylogenetic incongruence in an Asiatic species complex of the genus Caryodaphnopsis (Lauraceae)
Source: BMC Plant Biol. 2024 Jun 28;24:616. doi: 10.1186/s12870-024-05050-3 (PMC11212351; doi:10.1186/s12870-024-05050-3)

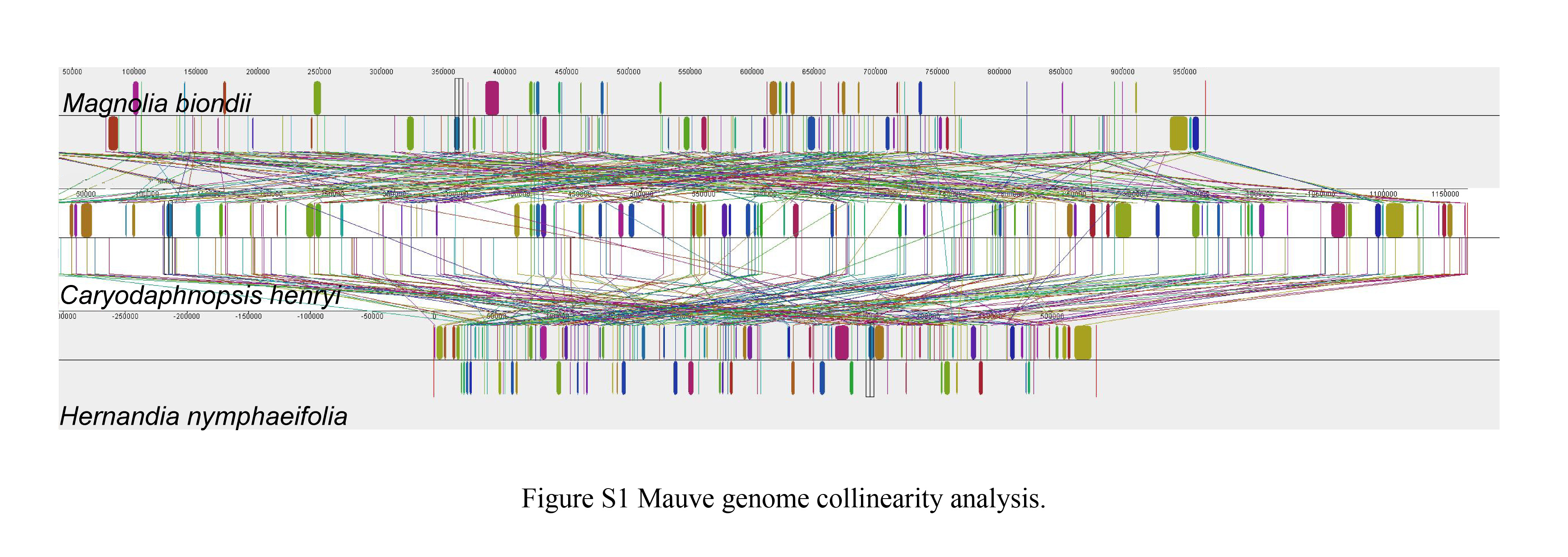

Supplement: Supplementary file 2 — Supplementary Material 2. [file 12870_2024_5050_MOESM2_ESM.jpeg]
